# Do randomized clinical trials with inadequate blinding report enhanced placebo effects for intervention groups and nocebo effects for placebo groups?

**DOI:** 10.1186/2046-4053-3-14

**Published:** 2014-02-21

**Authors:** Frederik Feys, Geertruida E Bekkering, Kavita Singh, Dirk Devroey

**Affiliations:** 1 Department of Family Medicine, Vrije Universiteit Brussel, Laarbeeklaan 103, Brussel B-1090, Belgium; 2 CEBAM Belgian Center of Evidence-based Medicine vzw, Leuven, Kapucijnenvoer 33, blok j, Leuven 3000, Belgium; 3 BeSyRe Bekkering Systematic Reviews, Geel, Belgium; 4 The Centre for Practice-Changing Research, Ottawa Hospital Research Institute, 501 Smyth Rd, Room L1247, Ottawa, Canada

**Keywords:** Placebo, Nocebo, Phosphodiesterase-5 inhibitor, Randomized controlled trial, Expectancy, Double blind, Bias, Treatment efficacy

## Abstract

**Background:**

Studies suggest that expectations powerfully shape clinical outcomes. For subjective outcomes in adequately blinded trials, health improvements are substantial and largely explained by non-specific factors.

The objective of this study was to investigate if unblinding in randomized controlled trials (RCTs) is associated with enhanced placebo effects for intervention groups and nocebo effects for placebo groups. For these effects, a secondary objective was to explore potential moderating factors.

**Methods:**

We included RCTs that investigated the efficacy of phosphodiesterase-5 (PDE-5) inhibitors for male erectile dysfunction by comparing one PDE-5 inhibitor to placebo. In addition, to be included studies must have reported scores for change from baseline, or baseline and final International Index of Erectile Functioning-Erectile Functioning domain score (IIEF-EF), and be published in either English, French, Dutch, or German.

We searched for both published and unpublished relevant trials using PUBMED, EMBASE, the Cochrane Central Register of Controlled Trials, a clinical trials register (clinicaltrials.gov) and the Food and Drug Administration clinical reviews through March 2012.

We evaluated the blinding status of trials with the Cochrane Risk of Bias Tool, using the domains of allocation sequence concealment, blinding of participants, healthcare providers and outcome assessors. Across these four domains, studies that scored low risk of bias were judged to be adequately blinded and studies that scored unclear or high risk of bias were judged to be inadequately blinded.

**Results:**

We included 110 studies (205 journal publications and 2 unpublished sources) that involved 23,877 participants; 93 (85%), 51 (46%), 93 (85%) and 93 (85%) studies were assessed with an unclear risk of bias for allocation concealment, blinding of participant, blinding of caregiver and blinding of outcome assessor, respectively. None of the studies reported testing of blinding.

None of the 205 journal publications provided sufficient details to assess allocation concealment, blinding of participants, caregivers and outcome assessors. After contacting authors for additional information, we judged five studies to be adequately (n = 1,202) and 16 to be inadequately (n = 3,006) blinded. The IIEF-EF score for placebo groups in adequately blinded trials versus inadequately blinded trials was 1.92 points (95% CI, 0.64 to 3.20) versus 1.56 (95% CI, 0.93 to 2.20), respectively. The IIEF-EF score for intervention groups in adequately blinded trials versus inadequately blinded trials was 9.40 (95% CI, 6.96 to 11.83) versus 8.33 (95% CI, 7.29 to 9.37), respectively. In a secondary analysis, prior experience with the drug affected the scores; in placebo groups with participants naïve to the intervention the score was 2.89 (95% CI, 2.33 to 3.45) versus -0.11 (95% CI, -2.06 to 1.84) with participants having prior experience. In the intervention groups, these scores were 7.99 (95% CI, 6.85 to 9.14) versus 8.33 (95% CI, 7.51 to 9.16), respectively.

Unblinding lowered placebo scores (creating a nocebo effect) by 19% (0.33 points; 95% CI, -0.96 to 1.62). Unblinding lowered intervention scores by 11% (1.0; 95% CI, -1.35 to 3.47). The results provided no conclusive evidence for nocebo or enhanced placebo effects. Patients taking a PDE-5 inhibitor for the first time experience a larger placebo effect that accounts for 35% of the total effect.

**Conclusions:**

Given the overall poor reporting of blinding in clinical trial reports and the small number of trials that could be rated as adequately or inadequately blinded, we could not draw any robust conclusions about the existence or absence of nocebo and enhanced placebo effects. A large placebo effect was found for patients taking PDE-5 inhibitors for the first time. It was not clear if previous exposure to the drug impacted trial blinding.

We found clear evidence that studies assessing a subjective continuous outcome fail to report on measures taken to secure double blinding. Although we observed a trend for the presence of a nocebo effect, there was insufficient evidence to quantify its impact on expectations. RCTs with patients with no prior experience with PDE-5 inhibitors reported larger placebo effects and possibly these studies were better blinded. Future research should further investigate the factors that contribute to blinding and their impact on health outcomes in randomized trials of subjectively assessed conditions. This research is part of a PhD project and has no external funding. The authors have no competing interests to declare.

## Background

A particular treatment effect may exert both non-specific and specific effects. A non-specific treatment effect is an outcome that does not arise according to an intended mechanism of action. This can be a response to placebo or reflection of spontaneous symptom improvement. A placebo is usually thought of as a sugar pill, but placebos can come in several forms; they may be things (syringes, medical devices), rituals (anamnesis, ingestion of drugs), places (hospital, doctor’s office), relationships (with doctor, self-help group) or medical beliefs to suggestive wordings [1]. The response to a placebo can be either positive for the outcome of interest, defined as a placebo effect, or negative for the outcome of interest, defined as a nocebo effect.

These effects are commonly explained by expectancy and conditional learning [2]. These two concepts overlap so, for convenience, we will use the term ‘expectancy’ to describe the mechanism behind placebo and nocebo effects.

Studies have shown that expectations can induce very powerful effects. In an experiment with an opioid painkiller, remifentanil, the presence of positive treatment expectancies was found to double the analgesic effect. Conversely, negative treatment expectancies interfered with the analgesic potential of the painkiller to the extent that the analgesic effect was completely abolished [3]. In a double-blind sham surgery trial, investigating a new surgical transplant technique for treatment of Parkinson’s disease, sham and real surgery interventions were equally effective. However, participants who thought they received the transplant reported better quality of life [4]. It seems that positive expectations were triggered by the perceived benefit from the treatment.

To control for the effects of expectations, the double-blind randomized controlled trial (RCT) design is commonly employed to study a novel intervention for its specific effects. Because neither participants nor investigators know who gets the intervention or the placebo, expectancies are balanced across groups. Double blinding makes groups comparable so that specific and non-specific treatment effects (that is, the effect size of the placebo group) can be ascertained with less potential for bias. Both intervention and placebo groups may have two important expectations in common: ‘I get the intervention or the placebo’ and ‘the intervention under study can cure my problem’.

However, there is little evidence that RCTs are, in fact, double-blinded [5]. Many factors can undermine double-blinded methodology, including poor randomization methods, imperfect concealment of allocation, and the use of a placebo that is distinguishable from the intervention. Furthermore, in RCTs of pharmacological agents, the presence of side effects may allow participants or investigators to guess correctly who has been allocated to intervention or placebo [6]. Therefore, the use of an active placebo that mimics some of the intervention’s side effects has been advocated to improve clinical trial blinding.

If an RCT is not adequately double-blinded, participants and investigators will know who gets what type of treatment. Expectations, therefore, could become unbalanced among treatment arms. A participant allocated to the intervention would have altered expectations: ‘I get the intervention’ and ‘the intervention under study can cure my problem.’ This enhances the participant’s prior expectations and can generate an enhanced placebo effect. For participants receiving the placebo, expectations could be ‘I get the placebo’ and ‘the intervention under study can cure my problem.’ This can lower participants’ expectations and generate a nocebo effect.

This review tested the hypothesis that unblinding in RCTs is associated with enhanced placebo effects for intervention groups and nocebo effects for placebo groups. We investigated this research question by conducting a meta-epidemiological study of phosphodiesterase-5 (PDE-5) inhibitors. For many years, this treatment has been an established baseline treatment for erectile dysfunction (ED). Numerous trials, overviews, and systematic reviews provide evidence for the efficacy and safety of sildenafil, tadalafil, and vardenafil [7]. There is also a growing evidence base for the newer molecules mirodenafil, udenafil, lodenafil, and avanafil. The PDE-5 inhibitors have been tested in many different populations, including those with broad-spectrum and specific comorbid conditions. The role of treatment expectations is of particular relevance to these medications for several reasons. Firstly, the evidence for efficacy relies solely on subjectively assessed outcomes, such as self-administered questionnaires (International Index of Erectile Functioning (IIEF)), event logs, and a Global Efficacy Question (GEQ) [8]. RCTs that use these subjective outcome measures are especially vulnerable to unblinding: non-blinded RCTs report 25% higher estimates of treatment effects than their blinded counterparts [9]. Whether this can be explained by nocebo effects in placebo groups or enhanced placebo effects for intervention groups was not reported. Secondly, since PDE-5 inhibitors are a well-tolerated and effective treatment for ED, initial expectations to treat this common male sexual problem are high for doctors, patients, and drug companies. Lastly, as suggestion can create expectancies [10], the domain of male sexual performance is a very suggestive domain, where expectations can play a fundamental role.

This meta-epidemiological study explored magnitude of expectancy and its moderating factors in RCTs. If the mechanisms moderating placebo effects such as expectations can be used in a non-deceptive way to produce clinically advantageous outcomes, then it may be possible to incorporate these mechanisms into evidence-based healthcare decision-making.

## Methods

We followed our published, open access pre-specified protocol during the systematic review [11]. A detailed methodology can be found as a supplemental file. We updated a dataset from an earlier published systematic review on PDE-5 inhibitors [12]. We restricted our search to English, French, Dutch, or German reports from the three agents approved by the Food and Drug Administration (FDA): sildenafil, vardenafil, and tadalafil.

Our search strategy employed a combination of controlled vocabulary (MeSH terms) and free text words. Three concepts were combined (AND): the intervention concept ‘PDE-5 inhibitor’, the disease concept ‘erectile dysfunction,’ and the design concept ‘RCT’. Strategies were adapted to search individual electronic databases. The last search was performed on 23 March 2012.

One reviewer (FF) manually screened the titles and abstracts. The full texts of potentially relevant reports were obtained. Multiple reports for the same study were linked to determine eligibility. Two reviewers (FF, GEB) screened full, unblinded texts. Disagreements were resolved by discussion.

We included RCTs that compared one PDE-5 inhibitor to placebo to investigate the efficacy of PDE-5 inhibitors in treating male ED. Studies had to report scores for change from baseline, or baseline and final IIEF erectile functioning domain (EF).

We used pilot-tested, electronic forms to extract data and assess risk of bias (ROB) of all included study reports. Two reviewers (FF, GEB) assessed blinding using four domains of the Cochrane’s ‘risk-of-bias’ tool that relate to blinding: allocation concealment, blinding of patient, blinding of caregiver, and blinding of outcome assessor [13]. In addition, the assessment included an evaluation of sequence generation, intention-to-treat (ITT) analysis and comparability of intervention and placebo groups. For each included study, we rated the ROB domains as low, high, or unclear. Disagreements were resolved by discussion.

To assess effect sizes, the most commonly used continuous subjective outcome in efficacy studies of PDE-5 inhibitors was used, the International Index of Erectile Functioning-Erectile Functioning domain score (IIEF-EF) [14]. We also assessed a dichotomous subjective outcome, the GEQ, to confirm consistency of the effect.

As a secondary objective, we explored moderating variables of placebo and intervention effect estimates that may explain enhanced placebo and nocebo effects. Of particular interest were prior experience with medication, drug side effects, study run-in period with placebo, sample size, geographical location of the study, single- or multi-center study, parallel or cross-over study, proportion of psychogenic etiology, prostate cancer or spinal cord injury, funding source, publication year, baseline disease severity, disease duration, study duration, type of PDE-5 inhibitor, commercial funding and number of follow-ups after baseline assessment. Rationales for choosing these moderators can be found in our published protocol.

For the treatment effect size, the change from baseline IIEF-EF score for intervention group and placebo group were used. If only final and baseline IIEF-EF scores were reported, we assumed change scores to be final-minus-baseline scores.

For placebo and intervention groups separately, we used a dichotomous subjective outcome (GEQ) and the two most common adverse events (AEs) reported (headache and flushing). We calculated risk ratios with 95% CIs. For GEQ, all randomized participants were included in the analysis, irrespective of how the authors of the report defined their ITT sample.

We imputed missing data, such as standard deviation, based on other available data, such as standard error, 95% CI, *t* value, or *P* value. If imputation of missing data was not possible, we contacted the original investigators to request missing data. If there was no response, we used data from matched studies.

We performed meta-analysis on studies using generic inverse variance. We used a random-effects model because the included studies showed considerable clinical (broad-spectrum and specific comorbid populations; different PDE-5 inhibitors) and methodological (study design, ROB) heterogeneity. The analysis included all parallel RCTs and a separate analysis included crossover RCTs. Regression analysis showed uncertain, very small effect size differences between crossover and parallel studies, so we decided to pool data from both study designs.

For every individual ROB domain, we grouped studies with low ROB and studies with unclear or high ROB. Studies that have a low ROB across all four ROB domains were considered adequately blinded. Studies that have a high ROB in at least one ROB domain or studies that have an unclear ROB across all four ROB domains were considered inadequately blinded. Adequately blinded studies were pooled and compared with inadequately blinded studies. For both groups, we calculated pooled intervention and placebo effect. We quantified the magnitude of enhanced placebo effects as the difference in intervention effect estimates among studies with inadequate blinding and studies with adequate blinding. Similarly, the magnitude of nocebo effects was quantified as the difference in placebo effect estimates among studies with inadequate and adequate blinding. Using RevMan, between-meta-analysis heterogeneity variance was calculated to express the variability in bias with *P* value and identified visually using a forest plot [15]. The magnitude of heterogeneity was assessed by calculating I^2^ and confirmed with a chi-square test. Blinding status is likely to be associated with other variables that also influence within-group intervention and placebo effect estimates. Using meta-regression analysis, we investigated baseline ED severity and publication year as possible confounders.

Inverse variance weighted regression analysis was done using Wilson macros (David B. Wilson, 4400 University Drive, MS 4 F4 Fairfax, VA 22030, United States) and SPSS 20 (IBM, 1 New Orchard Road, Armonk, New York 10504-1722, United States) to measure the impact of study, intervention, and patient factors on placebo and intervention effects separately [16]. A restricted maximum likelihood random effects variance component model was chosen to allow for residual variance. For multiple regression, a forward stepwise strategy with a lax entry criterion was used. Differences between groups were quantified with a 95% CI and then qualified statistically using a *t* test of no difference with *P* value. We had many studies with missing SDs. Therefore, analyzed groups of studies included RCTs with reported SD, and a random set of studies with missing SD was allowed up to 33% of the total sample. SDs were substituted with SDs from matched studies on sample size.

## Results

**Figure 1:**
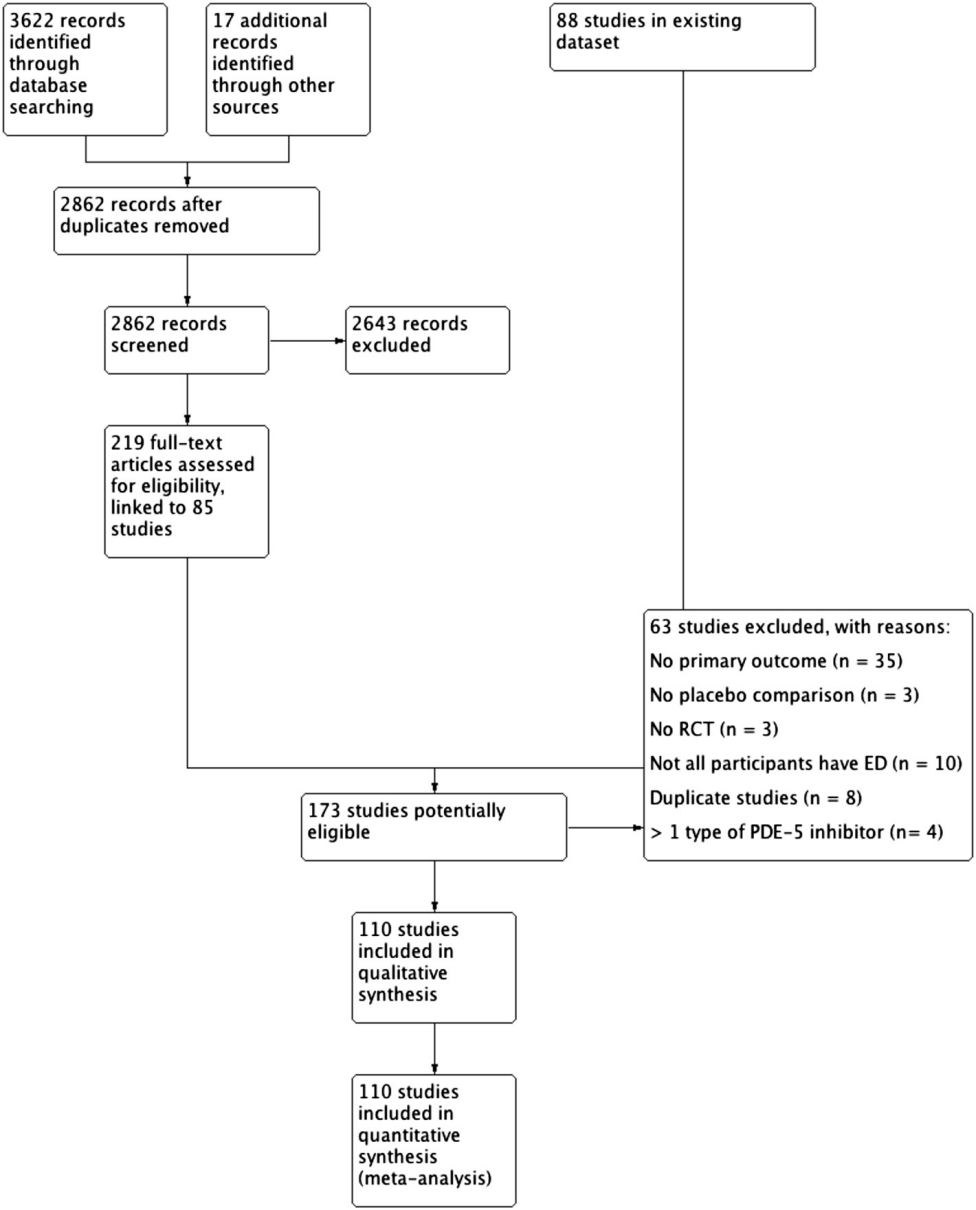
**PRISMA study flow diagram.** ED, erectile dysfunction; PDE-5, phosphodiesterase-5; RCT, randomized controlled trial.

We retrieved 3,622 records by electronic database searches. From our previous study we included 17 additional records that met screening criteria. After linking reports to individual studies, 173 studies were considered as potentially eligible. There was good agreement among reviewers as to what studies to include and exclude (Kappa statistic 0.85). An overview can be found in the PRISMA study flow diagram (Figure 1).

We excluded 63 studies, mostly because the primary outcome (IIEF-EF score) was missing (see Additional file 1). Thus, we included 110 studies (see Additional file 2). Of these, 107 studies were publicized in 205 journal publications, and three studies were not publicized (two in FDA records and one in ClinicalTrials.gov). Furthermore, 98 (89%) were parallel designed, 89 (81%) were commercially funded, 55 (50%) tested sildenafil, 10 (9%) included only patients naïve to the intervention, 40 (36%) reported no information on the most common AEs, 65 (59%) reported no information on the methods used to monitor AEs, and 69 (63%) reported no information on effect size variability, such as SDs or CIs.

Sixty-eight (62%) studies investigated broad spectrum ED (Table 1). Other populations included men with diabetes, prostatic cancer, depression, cardiovascular disease, renal failure, metabolic syndrome, spinal cord injury and post-traumatic stress syndrome. Median sample size was 195 (10th to 90th percentile 54 to 385).

**Table 1:** Overview of characteristics of 110 included randomized clinical trials

**Characteristics**	
Median sample size (range)	195 (13-817)
Median year of publication (range)	2006 (1998-2012)
Parallel group design	98 (89)
Crossover group design	10 (9)
Study run-in phase with placebo reported	2 (2)
Commercial funding	89 (81)
Single center study	9 (8)
Continent:	
Across continents	24 (22)
Africa	1 (1)
Asia	20 (18)
Europe	16 (15)
North America	23 (21)
Oceania	2 (2)
South America	5 (5)
Type of PDE-5 inhibitor:	
Sildenafil	55 (50)
Vardenafil	28 (26)
Tadalafil	27 (25)
Condition studied:	
Broad spectrum	68 (62)
Cardiovascular disease	5 (5)
Depression	5 (5)
Diabetes	9 (8)
Prostatic cancer	6 (6)
Metabolic syndrome	3 (3)
Multiple sclerosis	2 (2)
Post-traumatic stress syndrome	2 (2)
Spinal cord injury	2 (2)
Renal failure	4 (4)
Other	4 (4)
**Risk of bias:**	
Random sequence generated adequately	
Low	22 (20)
Unclear	88 (80)
High	0 (0)
Allocation concealed adequately	
Low	16 (15)
Unclear	93 (85)
High	1 (1)
Participants blinded adequately	
Low	58 (53)
Unclear	51 (46)
High	1 (1)
Caregivers blinded adequately	
Low	17 (16)
Unclear	93 (85)
High	0 (0)
Outcome assessors blinded adequately	
Low	17 (16)
Unclear	93 (85)
High	0 (0)
Overall blinded adequately	
Yes	5 (5)
No	48 (44)
Unclear	57 (52)
**Other study methods:**	
ITT analysis	5 (5)
Balanced baseline prognostic factors	82 (75)
Naïve to intervention	10 (9)
**Outcomes**	
Dichotomeous outcome, GEQ reported	69 (63)
Most common AEs reported*	70 (64)
**Methods used to monitor AEs**	
Prospective or routine monitoring	22 (20)
Spontaneous reporting	13 (12)
Patient checklist, questionnaire or diary	4 (4)
Systematic survey of patients	1 (1)
Not clear	65 (59)

Values are shown as numbers (%) unless stated otherwise. *In the case of phosphodiesterase-5 (PDE-5) inhibitors, the most common adverse events (AEs) are headache and flushing. GEQ, Global Efficacy Question; ITT, intention to treat.

Of the studies, 93 (85%), 51 (46%), 93 (85%) and 93 (85%) were assessed with an unclear ROB for allocation concealment, blinding of participant, blinding of caregiver and blinding of outcome assessor, respectively. Zero (0%) studies reported testing of blinding. Of the 110 studies, 5 (5%) were considered to be adequately blinded, 48 (44%) to be inadequately blinded and 57 (52%) to have a low ROB for at least one of four blinding-related domains. Of 108 requests to authors, 10 (9%) provided additional information on study methodology or primary outcomes with estimates of variability.

### Nocebo effects

**Figure 2:**
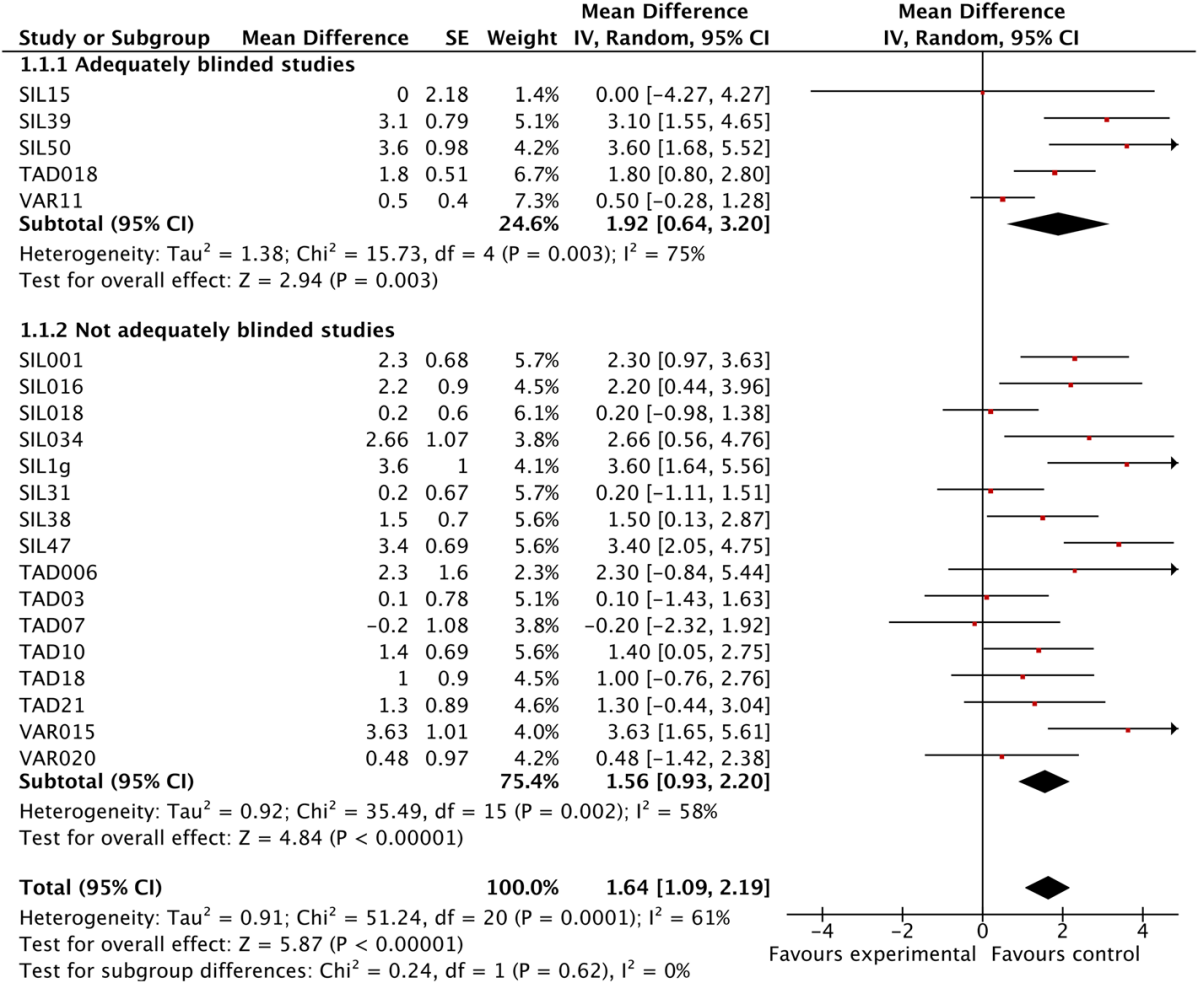
Forest plots with International Index of Erectile Functioning-Erectile Functioning domain (IIEF-EF) score in placebo groups of adequately blinded trials versus inadequately blinded trials.

Of the 48 inadequately blinded studies, we could calculate SDs for 13 studies and randomly added three more studies with matched SDs. The characteristics and ROB assessments of the studies in the primary analysis can be found in Additional files 3 and 4. The IIEF-EF domain score in placebo groups of five adequately blinded trials versus 16 inadequately blinded trials was 1.92 points (95% CI, 0.64 to 3.20) versus 1.56 (95% CI, 0.93 to 2.20), respectively. Unblinding lowered placebo scores (creating a nocebo effect) by 19% (0.33 points; 95% CI, -0.96 to 1.62) with moderate heterogeneity (I^2^ = 61%, *P* < 0.001; Table 2). Within-meta-analysis heterogeneity was moderate for inadequately (I^2^ = 58%, *P* = 0.002) and substantial for adequately (I^2^ = 75%, *P* = 0.003; Figure 2) blinded studies. One study (VAR11) accounted for 49% of heterogeneity. In this 4-week study, participants had 33% chance of receiving placebo and 72% had prior experience with the intervention. Another study (SIL15) was underpowered and mean effect sizes were calculated from skewed data.

**Table 2:** Enhanced placebo effects and nocebo effects

	**IIEF-EF scores for placebo groups**					**IIEF-EF scores for intervention groups**		
**ROB domain**	No. of studies (no. of participants)*	**Studies with low ROB (95% CI) (IV)**	**Studies with unclear or high ROB (95% CI) (IV)**	**Difference**^**a**^**(95% CI)**	**Between MA-homogeneity Q (*P* value)**	**Studies with low ROB (95% CI) (IV)**	**Studies with unclear or high ROB (95% CI) (IV)**	**Difference**^**b**^**(95% CI)**
Summary 4 ROB-domains	5 (1,202)/16 (3,006)	1.92 (0.64 to 3.20)	1.56 (0.93 to 2.20)	0.33 (-0.96 to 1.62)	20 (0.48)	9.40 (6.96 to 11.83)	8.33 (7.29 to 9.37)	-1.0 (-1.35 to 3.47)
Allocation concealment	13 (2,487)/48 (11,169)	1.82 (1.14 to 2.50)	1.75 (1.37 to 2.12)	0.07 (-0.8 to 0.93)	58 (0.88)	9.10 (7.94 to 10.26)	8.34 (7.63 to 9.05)	0.78 (-0.65 to 2.20)
Blinding participant	42 (9,159)/19 (3,442)	1.88 (1.45 to 2.31)	1.43 (0.95 to 1.91)	0.42 (-0.33 to 1.16)	64 (0.35)	8.39 (7.69 to 9.10)	8.23 (7.28 to 9.19)	0.16 (-1.02 to1.33)
Blinding caregiver	17 (3,652)/42 (8,652)	1.94 (1.18 to 2.69)	1.77 (1.40 to 2.15)	0.17 (-0.61 to 0.96)	56 (0.54)	8.46 (6.92 to 10.01)	8.64 (8.15 to 9.13)	-0.25 (-1.50 to 1.00)
Blinding outcome assessor	17 (3,278)/42 (9,659)	1.82 (0.99 to 2.65)	1.84 (1.48 to 2.21)	-0.02 (-0.83 to 0.79)	57 (0.52)	8.59 (7.15 to 10.03)	8.43 (7.80 to 9.06)	0.15 (-1.12 to 1.41)

Impact of unblinding on summary changes from baseline International Index of Erectile Functioning-Erectile Functioning domain (IIEF-EF) scores with 95% CI with estimates of nocebo^a,^ enhanced placebo^b^, placebo, and intervention effects. *Studies with low risk of bias (ROB) versus studies with unclear or high ROB. MA: Meta-Analysis, Q: Cochran’s Q, IV; Inverse Variance.

Using *post-hoc* multivariate regression analysis to explain nocebo effects, controlling for study blinding status (adequate or inadequate) (ß = -0.10, *P* = 0.819), studies with less participants naïve to the intervention (ß = 0.98, *P* = 0.022) showed smaller placebo effects. This model explained 95% of placebo scores.

### Enhanced placebo effects

**Figure 3:**
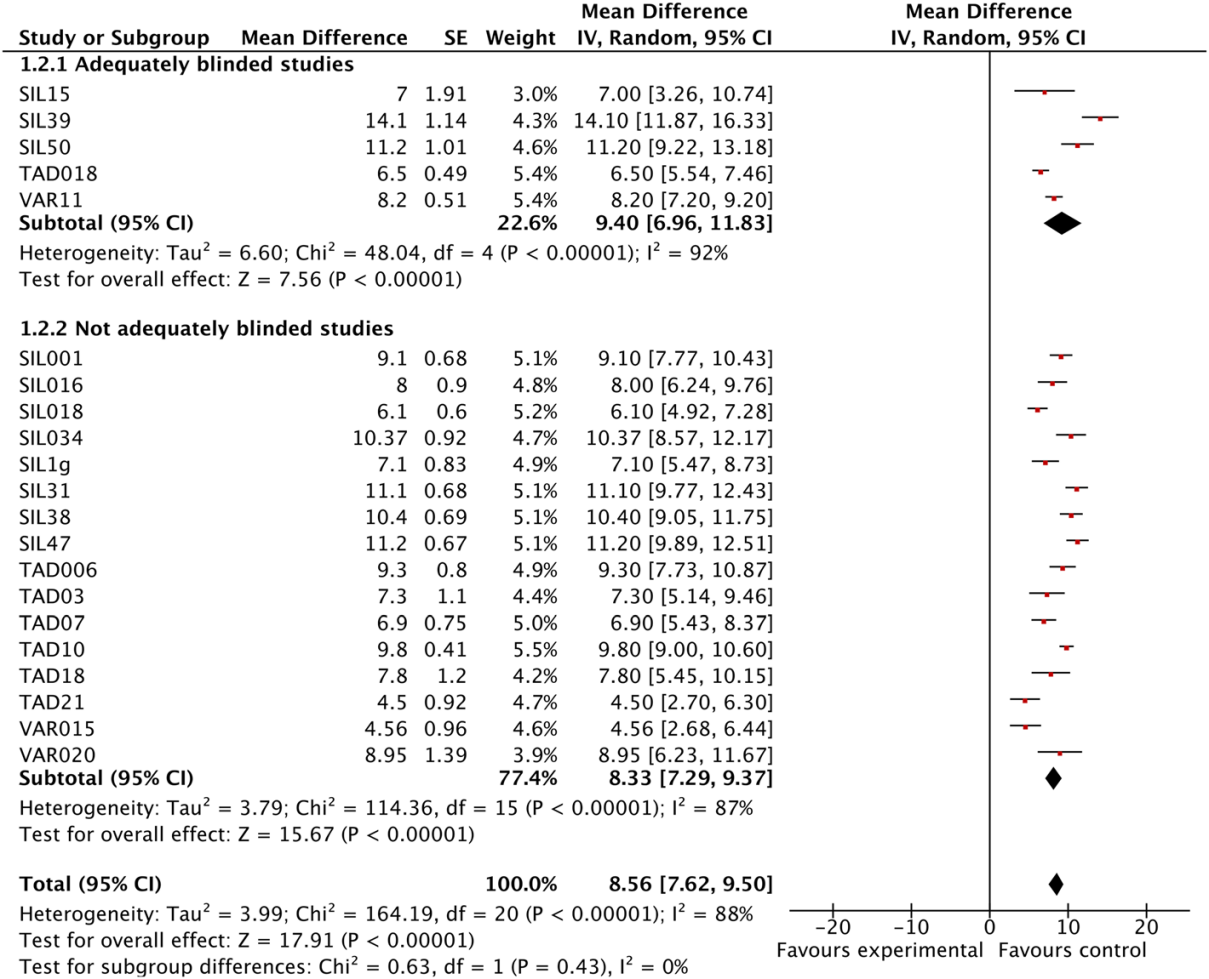
Forest plots with International Index of Erectile Functioning-Erectile Functioning domain (IIEF-EF) score in intervention groups of adequately blinded trials versus inadequately blinded trials.

The intervention effect in five adequately blinded trials versus 16 inadequately blinded trials was 9.40 (95% CI, 6.96 to 11.83) versus 8.33 (95% CI, 7.29 to 9.37), respectively. Unblinding lowered intervention scores by 11% (1.0 points; 95% CI, -1.35 to 3.47) with considerable heterogeneity (I^2^ = 88%, *P* < 0.001). Within-meta-analysis heterogeneity was considerable for inadequately (I^2^ = 87%, *P* < 0.001) and adequately (I^2^ = 92%, *P* < 0.001; Figure 3) blinded studies.

Using *post-hoc* multivariate regression analysis to explain intervention effects, controlling for study blinding status (adequate or inadequate) (ß = -0.27, *P* = 0.025), studies with larger placebo effects (ß = 0.45, *P* = 0.001) and more participants experiencing flushing AEs (ß = 0.73, *P* <0.001) showed larger intervention effects. This model explained 83% of intervention scores.

### Moderators of placebo effects

Using multivariate regression analysis to explain placebo effects, studies with participants naïve to the intervention (ß = 0.52, *P* = 0.012) and a higher proportion of participants randomized but not analyzed (ß = 0.43, *P* = 0.033) showed larger placebo effects. This model explained 79% of placebo scores (Table 3).

**Table 3:** Factors explaining placebo and intervention effects

**Univariate**	**No. of RCTs**	ß (*P*-value) on placebo effect*	ß (*P*-value) on intervention effect*
Adverse events:			
% Headache in placebo group	48	-0.11 (0.469)	0.02 (0.918)
% Headache in intervention group	48	-0.28 (0.052)	-0.11 (0.453)
Risk ratio for headache	48	-0.08 (0.569)	-0.11 (0.472)
% Flushing in placebo group	37	0.24 (0.135)	0.02 (0.917)
% Flushing in intervention group	37	0.10 (0.559)	0.30 (0.071)
Risk ratio for flushing	37	-0.17 (0.278)	0.21 (0.207)
Study related:			
Many follow-ups (≥4 vs 1 or 2)	21	0.05 (0.823)	-0.04 (0.867)
Sample size	61	-0.012 (0.928)	0.21 (0.098)
Long duration of double blind (>12 weeks versus ≤4 weeks)	15	0.56 (0.005)	0.52 (0.025)
% Randomized not analyzed	43	0.43 (0.002)	0.25 (0.102)
ITT analysis (yes vs no)	51	-0.07 (0.638)	-0.23 (0.094)
Publication year	61	0.02 (0.881)	-0.42 (<0.001)
Parallel study design (yes vs no)	61	-0.10 (0.451)	-0.07 (0.589)
Study run-in placebo	2	NA	NA
Commercial funding (yes vs no)	36	-0.09 (0.594)	-0.04 (834)
Continent (North America = ref)**	47		
Across continents	19	0.36 (0.038)	0.16 (0.392)
Asia	6	0.42 (0.005)	-0.07 (0.667)
Europe	9	0.25 (0.137)	0.21 (0.249)
Single center study (yes vs no)	56	0.14 (0.308)	-0.07 (0.623)
Type of PDE-5 inhibitor (tadalafil = ref)	59		
Sildenafil	30	0.24 (0.105)	0.11 (0.474)
Vardenafil	10	0.17 (0.259)	0.19 (0.199)
% Prior experience with intervention	14	-0.55 (0.013)	-0.05 (0.874)
Patient related:			
Prior experience with intervention (yes vs no)	9	-0.86 (<0.001)	-0.10 (0.772)
% Psychogenic etiology	40	0.11 (0.485)	-0.23 (0.150)
Prostate cancer or spinal cord injury (yes vs no)	19	0.04 (0.854)	-0.59 (0.003)
Baseline disease severity***	58	-0.31 (<0.001)	-0.44 (<0.001)
Disease duration	29	0.06 (0.761)	-0.07 (0.714)
**Multivariate**	**No. of RCTs**	**ß (*P*-value)**	
**Model on placebo effect (76% explained)**	10****		
Naïve to intervention (yes vs no)		0.52 (0.012)	
% Randomized not analyzed in placebo groups		0.44 (0.033)	
**Model on intervention effect (40% explained)**	107		
Placebo effect		0.32 (<0.001)	
Baseline disease severity***		-0.33 (<0.001)	
Year of publication		-0.26 (0.002)	
Sample size		0.23 (0.003)	

*Negative values represent lowering effects. **Analyses shown only for continents with more than two studies. ***Higher values on the International Index of Erectile Functioning-Erectile Functioning domain score questionnaire represent less severe cases of erectile dysfunction. ****Ten studies reported investigation of participants with prior experience or naïve to intervention. ß, standardized regression coëfficient; ITT, intention-to-treat; NA, not assessable or no data; PDE-5, phosphodiesterase-5; RCT, randomized controlled trial.

Univariate analysis revealed several patient- and study-related factors that predicted placebo scores: no prior experience with intervention (ß = -0.86, *P* < 0.001), more participants with prior experience of intervention (ß = -0.55, *P* = 0.013), more severe cases of ED (ß = -0.31, *P* <0.001), longer duration of studies (ß = 0.56, *P* = 0.005), more participants randomized but not analyzed (ß =0.43, *P* = 0.002), trials conducted in Asia (ß = 0.42, *P* = 0.005) and across continents (ß = 0.36, *P* = 0.038) have larger placebo effects.

More headache in the intervention group was associated with smaller placebo scores (ß = -0.28, *P* = 0.052). There was a trend towards more flushing in the placebo group with larger placebo scores (ß = 0.24, *P* = 0.135) and higher risk ratios of flushing with smaller placebo effects (ß = -0.17, *P* = 0.278).

### Moderators of intervention effects

Using multivariate regression analysis, studies with larger placebo scores (ß = 0.32, *P* < 0.001), more severe cases of ED (ß = -0.33, *P* < 0.001), older studies (ß = -0.26, *P* = 0.002) and larger sample sizes (ß = 0.23, *P* = 0.003) have larger intervention effects. This model explained 40% of intervention scores.

Using univariate regression analysis, more severe cases of ED (ß = -0.44, *P* < 0.001) and long duration of study double blindness (ß = 0.52, *P* = 0.025) showed larger intervention scores. Prostate cancer and spinal cord injury (ß = -0.59, *P* = 0.003), and later publication year (ß = -0.42, *P* < 0.001) showed smaller intervention scores.

More flushing (ß = 0.30, *P* = 0.071) and larger risk ratio for flushing (ß = 0.21, *P* = 0.207) in the intervention group was associated with larger effect sizes. The effect sizes are small to medium, but none of these were significant.

For all categorical moderators we performed subgroup meta-analyses. Forest plots provide absolute estimates of effects together with figures of heterogeneity (see Additional file 5). Reporting on methodological data was scarce so we did not have sufficient studies to present an adjusted effect estimate for enhanced placebo and nocebo effect, perform a confirmatory analysis of the primary hypothesis using a dichotomous outcome (GEQ), several preplanned sensitivity analyses, assess reporting bias visually, investigate AEs as possible moderators for enhanced placebo and nocebo effects, or assess the impact of using placebo during trial run-in periods.

A *post-hoc* multivariate regression analysis, using all the available evidence (110 studies) and with all four blinding related variables (allocation concealment, blinding of participant, caregiver or outcome assessor), found these factors did not influence placebo or intervention effects.

## Discussion

### Statement of principal findings

Our study found a consistent failure to report on study blinding in journal publications. Zero out of 205 publications (ignoring clinical trial and FDA records) provided sufficient detail to assess allocation concealment, blinding of participants, caregivers and outcome assessors. Only after contacting authors and skimming clinical trial register records, we qualified four and one study, respectively, as adequately blinded. Clearly, the blinding efforts for almost all trials remain unclear.

Our study found a small trend for unblinding in randomized clinical trials to underestimate placebo effects (creating a nocebo effect) and no evidence for the overestimation for intervention effects (enhanced placebo effects). Given the statistical uncertainty, lack of power and a large heterogeneity in the analysis, these findings should be interpreted as inconclusive (Table 2).

Secondary analyses provided evidence that prior experience with the intervention substantially lowered placebo scores, both expressed as a dichotomous (ß = -0.86, *P* < 0.001) and continuous (ß = -0.55, *P* = 0.013; Table 3) variable. It is not clear if participants with prior experience determine their allocation to placebo more efficiently and consequently engender lower expectancies as to their benefits. Intervention effect estimates were larger for trials that had larger placebo effects, included participants with more severe disease, for older trials and for larger trials. Whether these effects are related to enhanced expectations is not clear.

Some AEs may have a small effect on both placebo and intervention effect estimates, but findings were not consistent across all AEs and could not be confirmed statistically.

### Strengths and weaknesses of the study

Our research methods have been reported transparently and were open to public scrutiny. All analyses were pre-specified, were underpinned by scientific rationale and were performed using appropriate statistical methods. We found clear evidence that PDE-5 inhibitor studies for male ED that report a subjective continuous outcome systematically fail to report on measures taken to secure double blinding. In 1996, the CONSORT statement was publicly disseminated to improve reporting of clinical trials - 17 years later, we found that zero papers report on a recommended minimum set of blinding-related items [17]. Our review would have benefitted if reporting of methods in studies were better as this would have allowed us to evaluate the blinding status and perform meta-analyses. Only 5 of 110 studies provided some evidence as having a low ROB for allocation sequence concealment, blinding of participants, caregiver and outcome assessor. This small group of adequately blinded studies hampered a thorough analysis on the existence of nocebo or enhanced placebo effects. To assess how AEs may moderate outcomes was problematic; 36% of studies did not report the most common AEs and, in 59% of studies, the methods used for monitoring AEs were unclear. Also, for common, non-specific AEs, such as headache, it was difficult to assess whether reported events were due to the intervention or were present at baseline. The finding that intervention groups of studies reporting more participants experiencing headache also gives rise to smaller placebo effects is in concordance with our unblinding hypotheses. It may be that those allocated to the intervention become unblinded and consequently make it easier for study personnel to associate lack of headache with participants on placebo. Interpreting such interesting dynamics from our results is precarious and should only lead to hypothesis generation. Evidence points to prior experience with an intervention as a factor that substantially influences outcomes in RCTs. This study was restricted to the use of PDE-5 inhibitors in ED, an example of research using subjective outcomes that is particularly vulnerable to bias [9]. Clearly, the external validity of findings to different medical domains is limited. We performed many analyses and findings should be interpreted cautiously because of the risk of some false positive findings and confounding. As a final limitation of our study, we evaluated moderator effects within trial groups. Thus, we separately evaluated placebo and intervention groups and it follows that data were treated as from single-arm studies and we did not account for the fact that the analyzed data stem from RCTs. However, in our secondary multivariate analyses, explaining intervention effects, we included placebo group scores to account for randomization.

### Quality of the evidence

We included 110 studies that involved 23,877 analyzed participants. However, only five studies (n = 1,202) were adequately blinded. Due to poor reporting of SDs, only 16 inadequately (n = 3,006) blinded studies were analyzed. Using data from the systematic review of Turner and colleagues on the completeness of reporting for a random set of RCTs published in medical journals after the CONSORT statement [18], allocation concealment was roughly two times more likely to be reported adequately than in our publications, blinding of participants equally often, blinding of caregiver three times more often and blinding of outcome assessor two times more often.

Given the lack of adequately blinded studies and poor reporting of methodological information, our study cannot provide robust conclusions regarding the existence of nocebo or enhanced placebo effects in clinical trials of PDE-5 inhibitors. Mean baseline disease severity and publication year was comparable for both adequately and inadequately blinded studies and is assumed to not confound results. Seven studies included participants (n = 1,581) who used the drug for the first time, and in two studies (n = 675) all participants had prior experience with the intervention. Being naïve to the drug clearly effected placebo estimates, which was illustrated by subgroup analysis with non-overlapping CIs (95% CI, 2.33 to 3.45, heterogeneity 12%, and -2.06 to 1.84, heterogeneity 82%, respectively). The direction of the effect is in concordance with our hypothesis: people who use the drug for the first time may expect more and experience a larger placebo effect. Conversely, trial participants who have previous experience with the drug may be familiar with its adverse effects and may better distinguish intervention from placebo. For intervention estimates, CIs overlap (95% CI, 6.85 to 9.14, heterogeneity 84%, and 7.51 to 9.16, heterogeneity 0%, respectively) and suggest no direction of effect in concordance with our hypothesis. Overall, there is evidence that placebo effects are larger for groups of patients that use 3the drug for the first time. There is no evidence that patients who have previous experience report enhanced placebo effects.

### Potential biases in the review process

We prevented reviewer bias by using rigorous methods for searching, selecting studies, data collection and analysis. We published our study-protocol open to every reader and report on all pre-specified analyses. We performed a comprehensive search of the available evidence on a routinely measured subjective continuous outcome in ED. We excluded 63 (36%) studies because this outcome was not reported, suggesting that selective outcome reporting may be present. One out of four studies had baseline imbalance on outcome-related factors. It is unlikely that all the relevant studies were identified since half of all clinical trials that have been conducted and completed have never been published in academic journals, and trials with positive results are twice as likely to be published as others [19]. It is therefore expected that studies with negative findings (small treatment effects) due to low intervention and/or high placebo effects could not be included in our analyses and introduced selection bias. Missing data was also a problem within the available publications. During data collection, no publications provided sufficient details to assess study blinding. Only after a thorough quest, mainly after repeated contact with study authors, were 5 of 110 studies qualified as adequately blinded. Additional information from authors can reflect a response bias, as authors who have an interest in study blinding may be more willing to provide study data. Clearly, the blinding status for all but a few trials is unclear. Given the publication bias, selection bias and reporting bias, it is likely that we underestimated the nocebo and enhanced placebo effect due to unblinding in clinical trials.

### Agreements and disagreements with other studies or reviews

Methodological meta-epidemiological studies consistently find that inadequately blinded studies report overestimation of treatment benefits. Inadequate concealment of the sequence allocation, blinding of participants and outcome assessor is associated with 15%, 22% and 36% overestimation of treatment effects of subjective outcomes [20]. Whether these effects were moderated by placebo or intervention effects was not reported and therefore direct comparison with our results is not possible. An RCT for treating headache compared improvement rated both for blinded and non-blinded neurologists and patients. A very large nocebo and an enhanced placebo effect was reported by unblinded neurologists and patients. Moreover, their blinded counterparts show larger improvements on placebo than when on the active drug. Interestingly, in agreement with our hypothesis, neurologists may also have used presence or absence of side effects to identify treatment allocation [21]. Adequately blinded studies, defined as studies with confirmed prospectively measured blinding, show powerful placebo effects [22]. The use of a placebo inhaler for asthma symptoms resulted in subjective improvement, similar to the active drug [23]. In agreement with our findings, asthma treatment with enhanced expectations seems more powerful for placebo groups [24].

Previously, it was hypothesized that PDE-5 inhibitor-naïve participants may be more responsive to treatment [25]. Our findings could not confirm this. We found intervention effects to be roughly equivalent to those studies where participants were not naïve to the intervention. In agreement with our findings, previous experience with an intervention for restless leg syndrome showed smaller placebo effects, independently from baseline severity scores, but did not modify intervention effects [26]. It is widely accepted that placebo effects and baseline severity underpin intervention effects. For PDE-5 inhibitors, a meta-regression study of treatment effect modifiers in 13 sildenafil RCTs confirmed this [27]. We could not replicate their finding that ED duration influences treatment outcome. In agreement with the study, we observed that publications of more recent trials show smaller intervention effects. The apparent fading of reported intervention effectiveness was also found in a comprehensive systematic review by Gehr and colleagues [28]. Baseline disease was found to be the main factor contributing to this effect whereas sample size did not [28].

### Meaning of the study: possible explanations and implications for clinicians and policymakers

The reasons why publications fail to report blinding-related information will vary widely. Common sense would conclude that poor reporting of blinding is a reflection of the true absence of blinding. Evidence shows that correctly blinded studies with subjective, patient reported outcomes typically show large placebo effects. To demonstrate that an intervention is effective using a double blinded methodology, there is interest to lower placebo effects and thereby maximize perceived treatment effects (intervention minus placebo effect). In a broader perspective, poor reporting may also indicate a lack of interest from professional bodies such as ethical committees, journal editors, regulatory agencies and peer reviewers to insist on the importance of adequate reporting.

Clinicians have a duty to communicate the benefits and harms of PDE-5 inhibitors to their patients suffering with ED - scientists can help find and disseminate these answers by conducting methodologically sound studies and comprehensively reporting the methods and results of these studies in publications. Currently, poor reporting has left clinicians and scientists in the dark, thus holding back evidence-based progress. Reporting needs vast improvements, specifically on methods used for double blinding. Policy makers can improve this situation by endorsing legislation that requires all trial results to be published with adequate minimal reporting as recommended by CONSORT.

Participants in trials who have previous experience with an intervention may have lower placebo scores because they can better detect their treatment allocation. Indeed, changed bodily cues means ‘worse outcome’ and can lessen their expectations. This can lead to overestimation of intervention benefits. Participants who are naïve to the intervention are expected to provide more accurate and reliable information on intervention benefits and harms. Efficacy data for first time users of a drug would also improve the external validity of trial findings. Drug approval authorities should require pharmaceutical companies to provide trial data from intervention naïve participants that both approximates real drug benefits more closely and reflect expected value in a common, clinical setting.

The reasons why intervention effects deteriorate in time with smaller sample sizes and lower baseline risk remain unclear. However, we did notice that more recent trials did include less severe cases of ED, suggesting baseline risk as a moderating factor.

## Conclusions

Our research found a consistent failure to report on study blinding in journal publications. Poor reporting made it impossible to assess if studies were indeed double blinded and to quantify unblinding effects. It is naïve to assume that a RCT can separate placebo from real intervention effects, especially for subjective patient-reported outcomes. Traditional research focus lies on treatment effects (intervention minus placebo effect), but loses sight when it comes to underlying non-specific treatment effects. The current mantra ‘does this work’ should be complemented with ‘what does work’. Also, it is an unresolved question whether effectiveness results in unblinding or unblinding results in effectiveness [29]. CONSORT therefore took the decision that it is no longer required to report on the success of the double-blind procedure. We feel it withdrew us from the possibility to more profoundly understand how expectations shape clinical outcomes. Research should be aimed at understanding what patient- and trial-related factors impact blinding and how this subsequently impacts outcomes. A systematic review could assess across a range of medical domains how participants perceived treatment group allocation affects their estimates. It would be of interest to assess if AEs and previous experience provide clues to their perceived intervention.

## Abbreviations

AE: adverse event; ED: erectile dysfunction; EF: erectile functioning domain; FDA: Food and Drug Administration; GEQ: Global Efficacy Question; IIEF: International Index of Erectile Functioning; IIEF-EF: International Index of Erectile Functioning-Erectile Functioning domain score; ITT: intention-to-treat; PDE-5: phosphodiesterase-5; RCT: randomized controlled trial; ROB: risk of bias.

## Competing interests

The authors declare that they have no competing interests.

## Authors’ contributions

FF has the main responsibility of this review and drafted the protocol, developed the search strategy, searched for studies, extracted data into Review Manager and SPSS, carried out the analysis, interpreted the analysis, and drafted the final write-up of the review. FF and GEB applied inclusion criteria, and FF, GEB, and KS extracted data from studies. GEB, KS and DD gave feedback on subsequent protocol drafts and the publication manuscript. KS assisted with the preparation of the protocol manuscript. GEB and DD assisted where needed during the whole review process. All authors read and approved the final manuscript.

## Supplementary Material

Additional file 1 Characteristics of excluded studies. Click here for file

Additional file 2 Characteristics of 110 included randomized clinical trials. Click here for file

Additional file 3 **Characteristics of 21 included studies in primary analysis.** Risk of bias, methods used for monitoring adverse events. Click here for file

Additional file 4 **Characteristics of 21 included studies in primary analysis.** Methods, participants, intervention and outcomes. Click here for file

Additional file 5 Subgroup meta-analyses with forest plots. Click here for file
