# Supplementary material for: Do randomized clinical trials with inadequate blinding report enhanced placebo effects for intervention groups and nocebo effects for placebo groups?
Source: Syst Rev. 2014 Feb 21;3:14. doi: 10.1186/2046-4053-3-14 (PMC3939643; doi:10.1186/2046-4053-3-14)
Supplement: Additional file 1 — Characteristics of excluded studies. [file 2046-4053-3-14-S1.doc]

# Characteristics of excluded studies

Applies to all studies from updated search, not studies from previous study [11].

*SIL005*

| Reason for exclusion | No placebo comparison |
| --- | --- |

*SIL008*

| Reason for exclusion | No RCT |
| --- | --- |

*SIL010*

| Reason for exclusion | Not all participants have ED |
| --- | --- |

*SIL012*

| Reason for exclusion | No IIEF-EF scores |
| --- | --- |

*SIL013*

| Reason for exclusion | Not all participants have ED |
| --- | --- |

*SIL015*

| Reason for exclusion | No placebo comparison |
| --- | --- |

*SIL019*

| Reason for exclusion | Not all participants have ED |
| --- | --- |

*SIL020*

| Reason for exclusion | No IIEF-EF scores |
| --- | --- |

*SIL029*

| Reason for exclusion | No RCT but 2 Pooled RCTs |
| --- | --- |

*SIL032*

| Reason for exclusion | No IIEF-EF scores |
| --- | --- |

*SIL033*

| Reason for exclusion | No IIEF-EF scores |
| --- | --- |

*SIL1h*

| Reason for exclusion | No IIEF-EF scores |
| --- | --- |

*SIL2c*

| Reason for exclusion | No IIEF-EF scores |
| --- | --- |

*SIL2d*

| Reason for exclusion | No IIEF-EF scores |
| --- | --- |

*TAD002*

| Reason for exclusion | No RCT |
| --- | --- |

*TAD011*

| Reason for exclusion | Not all participants have ED |
| --- | --- |

*TAD013*

| Reason for exclusion | No placebo comparison |
| --- | --- |

*TAD014*

| Reason for exclusion | No IIEF-EF scores |
| --- | --- |

*TAD016*

| Reason for exclusion | Not all participants have ED |
| --- | --- |

*TAD017*

| Reason for exclusion | No IIEF-EF scores |
| --- | --- |

*TAD019*

| Reason for exclusion | Not all participants have ED |
| --- | --- |

*TAD020*

| Reason for exclusion | Not all participants have ED |
| --- | --- |

*VAR010*

| Reason for exclusion | No IIEF-EF scores |
| --- | --- |

*VAR012*

| Reason for exclusion | Not all participants have ED |
| --- | --- |

*VAR013*

| Reason for exclusion | Not all participants have ED |
| --- | --- |

*VAR014*

| Reason for exclusion | No IIEF-EF scores |
| --- | --- |

*VAR016*

| Reason for exclusion | No IIEF-EF scores |
| --- | --- |

*VAR022a*

| Reason for exclusion | No IIEF-EF scores |
| --- | --- |

*VAR022b*

| Reason for exclusion | No IIEF-EF scores |
| --- | --- |
