# Supplementary material for: Do randomized clinical trials with inadequate blinding report enhanced placebo effects for intervention groups and nocebo effects for placebo groups?
Source: Syst Rev. 2014 Feb 21;3:14. doi: 10.1186/2046-4053-3-14 (PMC3939643; doi:10.1186/2046-4053-3-14)
Supplement: Additional file 2 — Characteristics of 110 included randomized clinical trials. [file 2046-4053-3-14-S2.pdf]

Characteristics of 110 included randomised clinical trials. Study ID with 2 digits represents a study from previous dataset [11].

Study ID	Publication year	Countries	ED population	Sample size	Random sequence generation	Allocation concealment	Blinding participants	Blinding caregiver	Blinding outcome assessor	Methods monitoring AEs	ITT	% randomized and analyzed	Naïve to intervention?	% Naïve to intervention
SIL001	2009	Brazil, Korea, Russia, Spain, Sweden	Broad spectrum	188	Unclear	Unclear	Unclear	Unclear	Unclear	Spontaneous reporting	No	96.9	Unclear	?
SIL002	2006	Australia, Brazil, Japan, Mexico	Other	282	Unclear	Unclear	Low	Low	Low	Unclear	No	93.7	Unclear	?
SIL003	2006	USA	Broad spectrum	227	Unclear	Unclear	Low	Low	Low	Unclear	No	88.7	Unclear	?
SIL004	2006	US, Germany, UK, Canada	Depression	142	Unclear	Unclear	Unclear	Unclear	Unclear	Spontaneous reporting	No	?	Unclear	?
SIL006	2006	Israel	Post Traumatic Stress Syndrome	21	Unclear	Unclear	Unclear	Unclear	Unclear	Unclear	No	76.8	Unclear	?
SIL007	2006	India	Renal failure	32	Unclear	Low	Low	Unclear	Unclear	Unclear	Yes	100	Unclear	?
SIL009	2007	Thailand, Malaysia, Singapore	Metabolic syndrome	151	Unclear	Unclear	Unclear	Unclear	Unclear	Prospective or routine monitoring	No	97.6	Unclear	?
SIL011	2007	USA	Broad spectrum	155	Unclear	Unclear	Low	Low	Low	Unclear	No	86.4	Unclear	?
SIL014	2007	USA	Broad spectrum	351	Unclear	Unclear	Low	Unclear	Unclear	Unclear	No	95.1	Unclear	?
SIL016	2008	? US	Broad spectrum	202	Unclear	Unclear	Unclear	Unclear	Unclear	Spontaneous reporting	No	96.7	Unclear	?
SIL017	2008	Brazil, Italy, Germany, Poland,Turkey	Broad spectrum	294	Unclear	Unclear	Low	Unclear	Unclear	Spontaneous reporting	No	95.8	Unclear	?
SIL018	2007	Unclear	Broad spectrum	346	Unclear	Unclear	Unclear	Unclear	Unclear	Unclear	No	98.6	Unclear	?

SIL022	2009	Spain, Russia, Finland, France, Italy, Poland, Sweden, United Kingdom	Broad spectrum	817	Unclear	Unclear	Low	Unclear	Unclear	Prospective or routine monitoring	No	96.1	Unclear	?
SIL023	2009	Iran, possibly others	Post Traumatic Stress Syndrome	266	Unclear	Unclear	Low	Low	Unclear	Spontaneous reporting	Yes	100	Unclear	?
SIL024	2009	Unclear	Multiple sclerosis	180	Unclear	Unclear	Low	Low	Unclear	Spontaneous reporting	No	88.7	Yes	100
SIL025	2009	Israel	Broad spectrum	53	Unclear	Unclear	Low	Unclear	Unclear	Unclear	No	88.8	Unclear	?
SIL026	2010	Canada	Broad spectrum	162	Unclear	Unclear	Low	Low	Low	Spontaneous reporting	No	?	Unclear	?
SIL027	2010	Iran	Renal failure	27	Low	Unclear	Low	Unclear	Low	Systematic survey of patients	Unclear	?	Unclear	?
SIL028	2010	New Zealand	Prostatic cancer	45	Low	Low	Low	Unclear	Low	Unclear	No	72	Unclear	?
SIL030	2010	Unclear	Other	266	Unclear	Unclear	Low	Unclear	Unclear	Unclear	No	87.2	Unclear	?
SIL031	2010	Unclear	Broad spectrum	194	Unclear	Unclear	Unclear	Unclear	Unclear	Unclear	Unclear	?	Unclear	?
SIL034	2011	? Canada	Depression	185	Unclear	Unclear	Unclear	Unclear	Unclear	Unclear	No	91.5	Unclear	?
SIL035a	2008	US	Broad spectrum	346	Unclear	Unclear	Low	Unclear	Unclear	Unclear	No	98.6	Unclear	?
SIL035b	2008	US	Broad spectrum	246	Unclear	Unclear	Low	Unclear	Unclear	Unclear	No	98.4	Unclear	?
SIL13	1999	Denmark, Ireland, Italy, Norway, Sweden, UK	Broad spectrum	237	Unclear	Unclear	Low	Unclear	Unclear	Spontaneous reporting	No	93.3	Unclear	?

SIL15	2002	Brazil	Renal failure	41	Low	Low	Low	Low	Low	Unclear	No	85.4	Unclear	?
SIL16	2003	? USA	Broad spectrum	223	Unclear	Unclear	Low	Unclear	Unclear	Unclear	No	97.8	No	0
SIL1b	2004	? Canada	Cardiovascular disease	17	Unclear	Unclear	High	Unclear	Unclear	Unclear	Unclear	?	Unclear	?
SIL1g	2004	Unclear	Broad spectrum	59	Unclear	Unclear	Unclear	Unclear	Unclear	Prospective or routine monitoring	Unclear	?	Unclear	?
SIL25	2000	Japan	Broad spectrum	118	Unclear	Unclear	Unclear	Unclear	Unclear	Unclear	No	90.2	Unclear	?
SIL2a	2001	Unclear	Prostatic cancer	60	Unclear	Unclear	Unclear	Unclear	Unclear	Patient checklist, questionnaire or diary	Yes	100	Yes	100
SIL2f	2004	Unclear	Renal failure	13	Unclear	Unclear	Unclear	Unclear	Unclear	Unclear	Unclear	?	Unclear	?
SIL31	1998	USA	Broad spectrum	270	Unclear	Unclear	Unclear	Unclear	Unclear	Unclear	No	82.1	Unclear	?
SIL34	2000	Malaysia, the Philippines, Singapore	Broad spectrum	246	Low	High	Low	Low	Low	Unclear	No	96.9	Unclear	?
SIL35	2001	Taiwan	Broad spectrum	220	Unclear	Unclear	Low	Unclear	Unclear	Unclear	No	?	Unclear	?
SIL36	2001	Denmark, Finland, France, Germany, Sweden, UK	Diabetes	196	Unclear	Unclear	Low	Unclear	Unclear	Unclear	No	89.1	Unclear	?
SIL37	2000	Belgium, France, Germany, Netherlands, UK	Broad spectrum	273	Unclear	Unclear	Low	Unclear	Unclear	Prospective or routine monitoring	No	86.6	Unclear	?
SIL38	2001	USA	Broad spectrum	239	Unclear	Unclear	Unclear	Unclear	Unclear	Unclear	No	96.8	Unclear	?
SIL39	2001	USA	Depression	136	Low	Low	Low	Low	Low	Unclear	No	89.5	Unclear	?

SIL40	2002	Argentina, Chile, Peru, Uruguay	Broad spectrum	131	Unclear	Unclear	Unclear	Unclear	Unclear	Unclear	No	?	Unclear	?
SIL41a	2002	USA	Broad spectrum	232	Unclear	Unclear	Low	Unclear	Unclear	Unclear	No	94.3	Yes	100
SIL41b	2002	USA	Broad spectrum	178	Unclear	Unclear	Low	Unclear	Unclear	Unclear	No	90.4	Yes	100
SIL42	2001	Brazil, Mexico	Broad spectrum	214	Unclear	Unclear	Low	Unclear	Unclear	Unclear	No	87.3	Unclear	?
SIL43	2002	Colombia, Ecuador, Venezuela	Broad spectrum	146	Unclear	Unclear	Low	Unclear	Unclear	Unclear	No	91.8	Unclear	?
SIL44	2002	? UK	Other	32	Low	Low	Low	Unclear	Unclear	Unclear	No	?	Unclear	?
SIL46	2003	Thailand	Broad spectrum	124	Unclear	Unclear	Low	Unclear	Unclear	Prospective or routine monitoring	No	?	Unclear	?
SIL47	2003	Egypt, South Africa	Broad spectrum	248	Unclear	Unclear	Unclear	Unclear	Unclear	Prospective or routine monitoring	No	97.6	Unclear	?
SIL48	2001	Argentina, Australia, Brazil, Canada, Italy, Spain, Thailand, Turkey	Diabetes	167	Low	Low	Low	Unclear	Unclear	Unclear	No	87.4	Unclear	?
SIL49	2003	Korea	Broad spectrum	131	Unclear	Unclear	Low	Unclear	Unclear	Prospective or routine monitoring	No	?	Unclear	?
SIL50	2002	Belgium, France, Germany, Italy, Spain	Depression	156	Low	Low	Low	Low	Low	Unclear	No	92.9	Unclear	?
SIL51	2004	USA	Cardiovascular disease	142	Low	Unclear	Unclear	Unclear	Unclear	Unclear	No	94	Yes	100
SIL53	2005	Brazil	Cardiovascular disease	87	Low	Unclear	Low	Unclear	Unclear	Patient checklist, questionnaire or	No	72.5	Unclear	?

										diary				
SIL57	2005	USA, Canada	Cardiovascular disease	130	Low	Unclear	Unclear	Unclear	Unclear	Unclear	No	94.9	Unclear	?
SIL68	2002	Spain	Diabetes	80	Unclear	Unclear	Unclear	Unclear	Unclear	Unclear	No	86.8	Unclear	?
SIL69	2001	China	Broad spectrum	619	Unclear	Unclear	Unclear	Unclear	Unclear	Unclear	No	?	Unclear	?
TAD001	2006	Korea	Broad spectrum	121	Unclear	Unclear	Unclear	Unclear	Unclear	Prospective or routine monitoring	No	?	Unclear	?
TAD003	2006	The Netherlands	Prostatic cancer	60	Unclear	Unclear	Low	Unclear	Unclear	Patient checklist, questionnaire or diary	Yes	100	Unclear	?
TAD004	2006	Japan	Broad spectrum	172	Unclear	Unclear	Low	Low	Low	Spontaneous reporting	No	?	Unclear	?
TAD005	2006	Argentina, Brazil, France, Germany, United Kingdom.	Broad spectrum	159	Low	Low	Unclear	Unclear	Unclear	Spontaneous reporting	No	?	Unclear	?
TAD006	2006	Egypt, Turkey	Broad spectrum	124	Unclear	Unclear	Unclear	Unclear	Unclear	Prospective or routine monitoring	No	92.7	Unclear	?
TAD007	2006	China, Indonesia, Malaysia, Philippines, Singapore	Broad spectrum	242	Unclear	Unclear	Unclear	Unclear	Unclear	Prospective or routine monitoring	No	?	Unclear	?
TAD008	2007	USA	Broad spectrum	189	Low	Unclear	Unclear	Unclear	Unclear	Unclear	No	99	Unclear	?
TAD009	2007	North America, Europe, Austalia	Diabetes	195	Unclear	Low	Unclear	Unclear	Unclear	Spontaneous reporting	No	98.5	Unclear	?
TAD01	2005	Canada	Broad spectrum	141	Unclear	Unclear	Low	Low	Low	Prospective or routine monitoring	No	94.5	No	34

TAD010	2007	France, Germany, Italy, Spain.	Spinal cord injury	184	Low	Unclear	Unclear	Unclear	Unclear	Spontaneous reporting	No	?	No	21
TAD012	2009	Austria, France, Germany, Mexico, USA	Broad spectrum	316	Low	Unclear	Low	Unclear	Unclear	Unclear	No	92.4	Unclear	?
TAD015	2011	Germany, Greece, Italy, Poland, Spain.	Broad spectrum	213	Unclear	Low	Low	Unclear	Unclear	Unclear	No	97.5	Yes	100
TAD018	2012	USA, Canada, France, Germany, Greece, Italy, Mexico, Portugal, Russian Federation	Prostatic cancer	393	Low	Low	Low	Low	Low	Unclear	No	96.3	No	72.3
TAD02	2000	Canada	Broad spectrum	155	Unclear	Unclear	Unclear	Unclear	Unclear	Unclear	Unclear	?	Unclear	?
TAD03	2002	Spain	Diabetes	143	Unclear	Unclear	Unclear	Unclear	Unclear	Unclear	Unclear	?	Unclear	?
TAD04	2001	Taiwan	Broad spectrum	130	Low	Unclear	Unclear	Unclear	Unclear	Unclear	No	?	No	46.5
TAD05	2005	Australia	Broad spectrum	133	Low	Unclear	Low	Unclear	Unclear	Unclear	No	95.7	Unclear	?
TAD06	2000	Argentina, Canada, Mexico	Broad spectrum	143	Unclear	Unclear	Unclear	Unclear	Unclear	Unclear	Unclear	?	Unclear	?
TAD07	2005	USA	Broad spectrum	183	Unclear	Unclear	Unclear	Unclear	Unclear	Prospective or routine monitoring	No	93.9	No	17
TAD08	2004	USA, Puerto Rico	Broad spectrum	205	Unclear	Unclear	Low	Low	Low	Unclear	No	?	No	19
TAD09	2004	UK, Italy	Broad spectrum	215	Unclear	Low	Unclear	Unclear	Unclear	Prospective or routine monitoring	No	?	No	51.4
TAD10	2004	Czech Republic, Poland, Slovakia, Israel, Hungary,	Broad spectrum	403	Unclear	Unclear	Unclear	Unclear	Unclear	Prospective or routine monitoring	No	98.4	No	46.4

		Lebanon, Romania												
TAD11	2006	China, Singapore, Philippines	Broad spectrum	247	Unclear	Unclear	Unclear	Unclear	Unclear	Prospective or routine monitoring	Unclear	?	Unclear	?
TAD13	2004	Canada	Broad spectrum	83	Unclear	Unclear	Low	Unclear	Unclear	Unclear	Unclear	?	Unclear	?
TAD16	2004	Canada, Germany, Italy, The Netherlands, Spain, UK, USA	Prostatic cancer	293	Low	Unclear	Low	Low	Low	Prospective or routine monitoring	No	96.3	Unclear	?
TAD18	2001	Unclear	Broad spectrum	71	Unclear	Unclear	Unclear	Unclear	Unclear	Unclear	Yes	100	Unclear	?
TAD21	2006	Unclear	Diabetes	198	Unclear	Unclear	Unclear	Unclear	Unclear	Unclear	Unclear	?	Unclear	?
VAR001	2006	Unclear	Broad spectrum	254	Unclear	Unclear	Unclear	Unclear	Unclear	Unclear	No	97.5	Yes	100
VAR002	2006	USA, Canada, Portugal, Italy, Spain, France, Australia, Hong Kong, Mexico.	Spinal cord injury	394	Unclear	Unclear	Unclear	Unclear	Unclear	Unclear	No	?	Unclear	?
VAR003	2006	Japan	Diabetes	441	Unclear	Low	Low	Unclear	Unclear	Unclear	No	97	Unclear	?
VAR004	2006	USA, Germany, Canada, Australia, Austria, Brazil, Greece, South Korea, Mexico, Sweden, Singapore, Turkey.	Broad spectrum	364	Unclear	Unclear	Unclear	Unclear	Unclear	Unclear	No	95	Unclear	?
VAR005	2006	USA, Canada, France, Spain, Italy.	Depression	260	Unclear	Unclear	Unclear	Unclear	Unclear	Unclear	No	92.9	Unclear	?
VAR006	2006	Germany	Diabetes	302	Unclear	Unclear	Low	Unclear	Unclear	Unclear	No	95	Yes	100

VAR007	2007	Spain	Broad spectrum	121	Unclear	Unclear	Low	Low	Low	Unclear	No	93.8	Unclear	?
VAR008	2007	UK	Broad spectrum	432	Unclear	Unclear	Unclear	Unclear	Unclear	Unclear	No	62.2	Unclear	?
VAR009	2008	Malaysia, Singapore, Thailand, the Philippines, Hong Kong, China, Indonesia	Broad spectrum	334	Unclear	Unclear	Unclear	Unclear	Unclear	Unclear	No	94.3	No	36
VAR01	2003	USA, Canada	Prostatic cancer	284	Low	Unclear	Low	Unclear	Unclear	Unclear	No	96.6	No	20
VAR011	2008	USA	Metabolic syndrome	385	Unclear	Low	Unclear	Unclear	Unclear	Unclear	No	?	No	20
VAR015	2009	Italy	Broad spectrum	46	Unclear	Unclear	Unclear	Unclear	Unclear	Unclear	Unclear	?	Yes	100
VAR017	2009	Unclear	Broad spectrum	175	Unclear	Unclear	Unclear	Unclear	Unclear	Unclear	No	87.1	Unclear	?
VAR018	2010	Belgium, France, Germany, the Netherlands, Spain, South Africa	Broad spectrum	355	Unclear	Unclear	Low	Unclear	Unclear	Prospective or routine monitoring	No	98.1	No	23.5
VAR019	2010	Australia, Canada, Mexico, USA	Broad spectrum	327	Unclear	Unclear	Low	Unclear	Unclear	Prospective or routine monitoring	No	96.5	No	16.9
VAR02	2002	USA, Canada	Broad spectrum	373	Unclear	Unclear	Unclear	Unclear	Unclear	Unclear	No	94.7	No	33
VAR020	2010	Unclear	Multiple sclerosis	21	Unclear	Unclear	Unclear	Unclear	Unclear	Unclear	Unclear	?	Unclear	?
VAR021	2011	Germany	Metabolic syndrome	144	Unclear	Unclear	Low	Unclear	Unclear	Prospective or routine monitoring	No	96	No	32
VAR04	2004	Japan	Broad spectrum	137	Unclear	Low	Low	Unclear	Unclear	Unclear	No	98.6	Unclear	?
VAR05	2004	Austria, France, Germany, Greece,	Broad spectrum	304	Unclear	Unclear	Low	Unclear	Unclear	Spontaneous	No	94.2	No	48

		Italy, Netherlands, Spain, Switzerland, UK								reporting				
VAR07	2004	North America, Europe, Asia-Pacific	Broad spectrum	452	Unclear	Unclear	Low	Unclear	Unclear	Prospective or routine monitoring	No	97.6	No	0
VAR09	2001	Belgium, France, Germany, Netherlands, Poland, Rep of South Africa, USA	Broad spectrum	294	Unclear	Unclear	Low	Unclear	Unclear	Patient checklist, questionnaire or diary	No	?	No	51
VAR10	2003	USA, Canada	Diabetes	277	Unclear	Unclear	Unclear	Unclear	Unclear	Prospective or routine monitoring	No	92.6	No	42.5
VAR11	2004	North America and Europe	Broad spectrum	476	Low	Low	Low	Low	Low	Prospective or routine monitoring	No	96.2	No	27.5
VAR12	2005	Europe, North America, South America, Asia-Pacific	Broad spectrum	509	Unclear	Unclear	Unclear	Unclear	Unclear	Unclear	No	97.3	No	12
VAR14	2005	? Canada, USA	Broad spectrum	225	Low	Unclear	Low	Unclear	Unclear	Unclear	No	98.3	No	14
VAR15	2005	Germany	Cardiovascular disease	354	Unclear	Unclear	Low	Unclear	Unclear	Unclear	No	91.2	Unclear	?
VAR19	2006	Unclear	Unclear	383	Unclear	Unclear	Unclear	Unclear	Unclear	Unclear	Unclear	?	Unclear	?
