# Supplementary material for: Do randomized clinical trials with inadequate blinding report enhanced placebo effects for intervention groups and nocebo effects for placebo groups?
Source: Syst Rev. 2014 Feb 21;3:14. doi: 10.1186/2046-4053-3-14 (PMC3939643; doi:10.1186/2046-4053-3-14)
Supplement: Additional file 5 — Subgroup meta-analyses with forest plots. [file 2046-4053-3-14-S5.pdf]

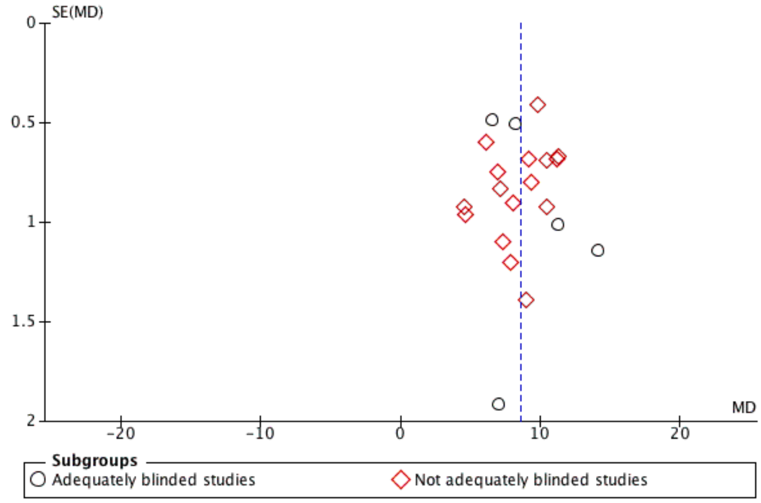

Funnel plot of comparison: 1 IIEF-EF change scores: blinding across 4 RoB domains, outcome: 1.2 Effect estimate intervention groups.

Figure 4 (Analysis 6.1)

Study or Subgroup	Mean Difference	SE	Weight	Mean Difference IV, Random, 95% CI
SIL001	2.3	0.67989	2.0%	2.30 [0.97, 3.63]
SIL002	5.2	0.663129	2.0%	5.20 [3.90, 6.50]
SIL003	3.6	0.714299	1.9%	3.60 [2.20, 5.00]
SIL006	6.38	0.966705	1.5%	6.38 [4.49, 8.27]
SIL007	0.3	1.753625	0.8%	0.30 [−3.14, 3.74]
SIL011	3.4	1.000253	1.5%	3.40 [1.44, 5.36]
SIL014	1.86	0.961774	1.5%	1.86 [−0.03, 3.75]
SIL016	2.2	0.899509	1.6%	2.20 [0.44, 3.96]
SIL017	1.4	0.599619	2.1%	1.40 [0.22, 2.58]
SIL018	0.2	0.599656	2.1%	0.20 [−0.98, 1.38]
SIL022	2.1	0.418374	2.4%	2.10 [1.28, 2.92]
SIL023	2.2	0.381528	2.5%	2.20 [1.45, 2.95]
SIL024	2.6	1.080105	1.4%	2.60 [0.48, 4.72]
SIL025	1.4	1.000253	1.5%	1.40 [−0.56, 3.36]
SIL026	−0.5	0.699906	2.0%	−0.50 [−1.87, 0.87]
SIL027	0.92	2.077352	0.6%	0.92 [−3.15, 4.99]
SIL028	1.24	1.247726	1.2%	1.24 [−1.21, 3.69]
SIL030	1.1	0.699719	2.0%	1.10 [−0.27, 2.47]
SIL034	2.66	1.069968	1.4%	2.66 [0.56, 4.76]
SIL15	0	2.18	0.5%	0.00 [−4.27, 4.27]
SIL16	−1.2	0.727055	1.9%	−1.20 [−2.63, 0.23]
SIL25	2.3	0.772015	1.8%	2.30 [0.79, 3.81]
SIL35	4.56	0.730352	1.9%	4.56 [3.13, 5.99]
SIL36	1.1	0.612152	2.1%	1.10 [−0.10, 2.30]
SIL40	1.38	0.741728	1.9%	1.38 [−0.07, 2.83]
SIL41b	2.9	0.683699	2.0%	2.90 [1.56, 4.24]
SIL44	1.7	1.270435	1.2%	1.70 [−0.79, 4.19]
SIL51	4.5	0.781353	1.8%	4.50 [2.97, 6.03]
SIL53	2.5	1.549244	0.9%	2.50 [−0.54, 5.54]
TAD005	0.87	0.771589	1.8%	0.87 [−0.64, 2.38]
TAD006	2.3	1.60068	0.9%	2.30 [−0.84, 5.44]
TAD009	1.3	0.612152	2.1%	1.30 [0.10, 2.50]
TAD01	−0.9	1.099852	1.4%	−0.90 [−3.06, 1.26]
TAD012	0.49	0.781353	1.8%	0.49 [−1.04, 2.02]
TAD015	3.3	0.730573	1.9%	3.30 [1.87, 4.73]
TAD018	1.8	0.51001	2.3%	1.80 [0.80, 2.80]
TAD03	0.1	0.783276	1.8%	0.10 [−1.44, 1.64]
TAD06	0.7	0.719907	1.9%	0.70 [−0.71, 2.11]
TAD07	−0.2	1.08075	1.4%	−0.20 [−2.32, 1.92]
TAD08	0.3	0.79963	1.8%	0.30 [−1.27, 1.87]
TAD09	0.4	0.942857	1.6%	0.40 [−1.45, 2.25]
TAD10	1.4	0.689143	2.0%	1.40 [0.05, 2.75]
TAD16	1.1	0.6153	2.1%	1.10 [−0.11, 2.31]
TAD18	1	0.899244	1.6%	1.00 [−0.76, 2.76]
VAR001	2.3	0.7475	1.9%	2.30 [0.83, 3.77]
VAR003	2.6	0.676015	2.0%	2.60 [1.28, 3.92]
VAR006	2.05	0.474335	2.4%	2.05 [1.12, 2.98]
VAR007	1.6	0.994066	1.5%	1.60 [−0.35, 3.55]
VAR015	3.63	4.850045	0.1%	3.63 [−5.88, 13.14]
VAR018	1.4	0.52307	2.3%	1.40 [0.37, 2.43]
VAR019	1.4	0.48541	2.3%	1.40 [0.45, 2.35]
VAR02	1.2	0.594552	2.1%	1.20 [0.03, 2.37]
VAR020	0.48	0.966705	1.5%	0.48 [−1.41, 2.37]
VAR04	3.1	0.783276	1.8%	3.10 [1.56, 4.64]
VAR05	1.5	0.469631	2.4%	1.50 [0.58, 2.42]
VAR11	0.5	0.403791	2.5%	0.50 [−0.29, 1.29]
VAR12	1.3	0.432945	2.4%	1.30 [0.45, 2.15]
**Total (95% CI)**			**100.0%**	**1.71 [1.36, 2.05]**

Heterogeneity: $Tau^2 = 1.13$; $Chi^2 = 184.01$, df = 56 ($P < 0.00001$); $I^2 = 70\%$
Test for overall effect: Z = 9.57 ($P < 0.00001$)

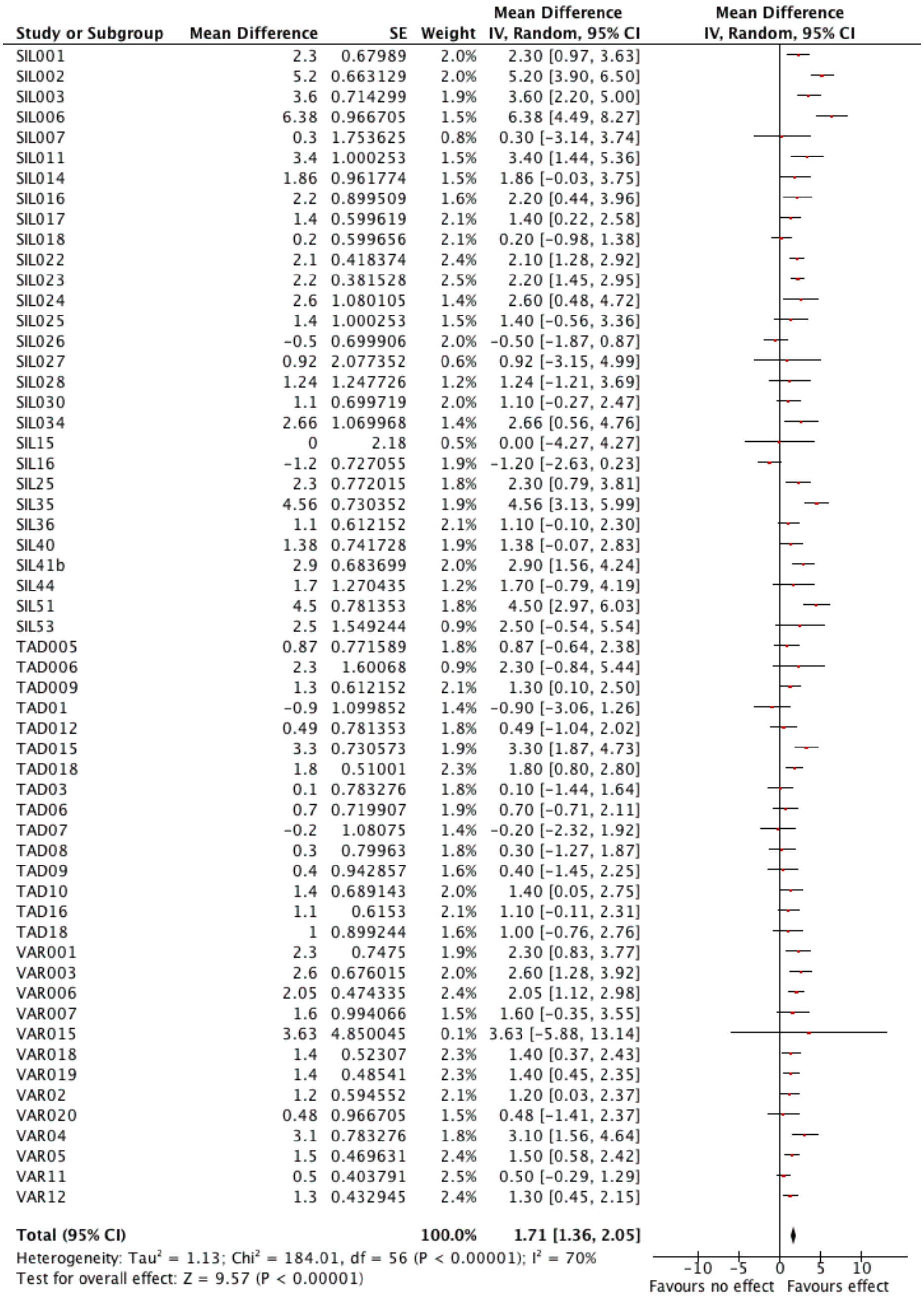

Forest plot of comparison: 6 IIEF-EF change scores: RCTs using parallel and crossover design, outcome: 6.1 Effect estimate placebo groups.

Figure 5 (Analysis 6.2)

Study or Subgroup	Mean Difference	SE	Weight	Mean Difference IV, Random, 95% CI	Mean Difference IV, Random, 95% CI
SIL001	9.1	0.679833	1.9%	9.10 [7.77, 10.43]	
SIL002	11.7	0.663333	1.9%	11.70 [10.40, 13.00]	
SIL003	9.3	0.714353	1.9%	9.30 [7.90, 10.70]	
SIL006	11.08	1.358089	1.5%	11.08 [8.42, 13.74]	
SIL007	9.3	1.753625	1.2%	9.30 [5.86, 12.74]	
SIL011	8.9	1.000203	1.7%	8.90 [6.94, 10.86]	
SIL014	9.17	0.979662	1.7%	9.17 [7.25, 11.09]	
SIL016	8	0.899606	1.8%	8.00 [6.24, 9.76]	
SIL017	8.7	0.599619	1.9%	8.70 [7.52, 9.88]	
SIL018	6.1	0.600082	1.9%	6.10 [4.92, 7.28]	
SIL022	9.5	0.316981	2.1%	9.50 [8.88, 10.12]	
SIL023	3.6	0.390199	2.0%	3.60 [2.84, 4.36]	
SIL024	7.2	0.750467	1.9%	7.20 [5.73, 8.67]	
SIL025	6.8	1.20049	1.6%	6.80 [4.45, 9.15]	
SIL026	2.1	0.599959	1.9%	2.10 [0.92, 3.28]	
SIL027	5.79	1.54477	1.3%	5.79 [2.76, 8.82]	
SIL028	6.39	1.187939	1.6%	6.39 [4.06, 8.72]	
SIL030	5.4	0.599587	1.9%	5.40 [4.22, 6.58]	
SIL034	10.37	0.920249	1.7%	10.37 [8.57, 12.17]	
SIL15	7	1.158283	1.6%	7.00 [4.73, 9.27]	
SIL16	8.4	0.714353	1.9%	8.40 [7.00, 9.80]	
SIL25	12.3	0.743194	1.9%	12.30 [10.84, 13.76]	
SIL35	10.66	0.720818	1.9%	10.66 [9.25, 12.07]	
SIL36	10	0.716198	1.9%	10.00 [8.60, 11.40]	
SIL40	5.98	0.73978	1.9%	5.98 [4.53, 7.43]	
SIL41b	9.7	0.713379	1.9%	9.70 [8.30, 11.10]	
SIL44	13.3	2.760809	0.7%	13.30 [7.89, 18.71]	
SIL51	9.5	0.837855	1.8%	9.50 [7.86, 11.14]	
SIL53	11.2	1.462624	1.4%	11.20 [8.33, 14.07]	
TAD005	9.35	0.804142	1.8%	9.35 [7.77, 10.93]	
TAD006	9.3	0.800167	1.8%	9.30 [7.73, 10.87]	
TAD009	4.5	0.71988	1.9%	4.50 [3.09, 5.91]	
TAD01	8	0.79949	1.8%	8.00 [6.43, 9.57]	
TAD012	8.03	0.448769	2.0%	8.03 [7.15, 8.91]	
TAD015	7.3	0.497391	2.0%	7.30 [6.33, 8.27]	
TAD018	6.5	0.4899	2.0%	6.50 [5.54, 7.46]	
TAD03	7.3	1.096016	1.6%	7.30 [5.15, 9.45]	
TAD06	5.6	1.012517	1.7%	5.60 [3.62, 7.58]	
TAD07	6.9	0.750126	1.9%	6.90 [5.43, 8.37]	
TAD08	9.3	0.600161	1.9%	9.30 [8.12, 10.48]	
TAD09	11.1	2.111129	1.0%	11.10 [6.96, 15.24]	
TAD10	9.8	0.408084	2.0%	9.80 [9.00, 10.60]	
TAD16	5.3	0.506429	2.0%	5.30 [4.31, 6.29]	
TAD18	7.8	1.2	1.6%	7.80 [5.45, 10.15]	
VAR001	8.8	0.436011	2.0%	8.80 [7.95, 9.65]	
VAR003	9	0.386822	2.0%	9.00 [8.24, 9.76]	
VAR006	7.79	0.634635	1.9%	7.79 [6.55, 9.03]	
VAR007	10.9	0.819436	1.8%	10.90 [9.29, 12.51]	
VAR015	4.56	4.589402	0.3%	4.56 [-4.44, 13.56]	
VAR018	8.6	0.547024	2.0%	8.60 [7.53, 9.67]	
VAR019	8.5	0.627571	1.9%	8.50 [7.27, 9.73]	
VAR02	9	0.562143	2.0%	9.00 [7.90, 10.10]	
VAR020	8.95	1.390048	1.4%	8.95 [6.23, 11.67]	
VAR04	12.6	1.144751	1.6%	12.60 [10.36, 14.84]	
VAR05	10.8	0.636719	1.9%	10.80 [9.55, 12.05]	
VAR11	8.2	0.50824	2.0%	8.20 [7.20, 9.20]	
VAR12	8.8	0.463406	2.0%	8.80 [7.89, 9.71]	
**Total (95% CI)**			**100.0%**	**8.36 [7.78, 8.94]**	

Heterogeneity: $Tau^2 = 4.13$; $Chi^2 = 582.42$, df = 56 ($P < 0.00001$); $I^2 = 90\%$

Test for overall effect: $Z = 28.36$ ($P < 0.00001$)

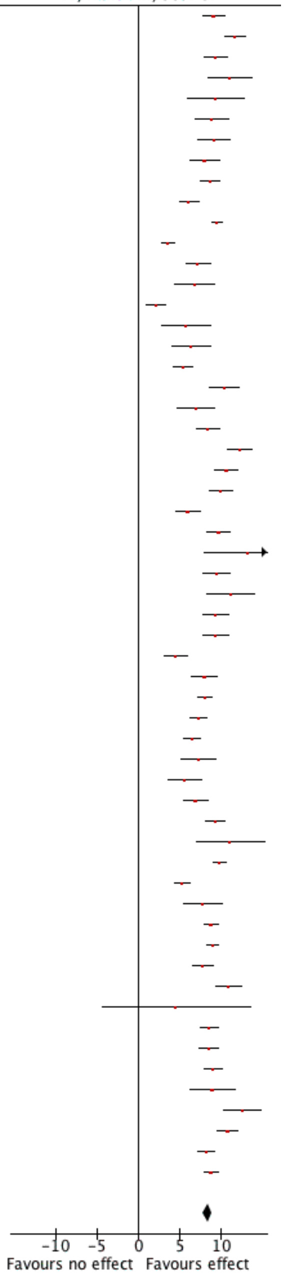

Forest plot of comparison: 6 IIEF-EF change scores: RCTs using parallel and crossover design, outcome: 6.2 Effect estimate intervention groups.

Figure 6 (Analysis 7.1)

Study or Subgroup	Mean Difference	SE	Weight	Mean Difference IV, Random, 95% CI
SIL001	2.3	0.67989	2.1%	2.30 [0.97, 3.63]
SIL002	5.2	0.663129	2.1%	5.20 [3.90, 6.50]
SIL003	3.6	0.714299	2.0%	3.60 [2.20, 5.00]
SIL011	3.4	1.000253	1.5%	3.40 [1.44, 5.36]
SIL014	1.86	0.961774	1.6%	1.86 [−0.03, 3.75]
SIL016	2.2	0.899509	1.7%	2.20 [0.44, 3.96]
SIL017	1.4	0.599619	2.3%	1.40 [0.22, 2.58]
SIL018	0.2	0.599656	2.3%	0.20 [−0.98, 1.38]
SIL022	2.1	0.418374	2.6%	2.10 [1.28, 2.92]
SIL023	2.2	0.381528	2.7%	2.20 [1.45, 2.95]
SIL024	2.6	1.080105	1.4%	2.60 [0.48, 4.72]
SIL025	1.4	1.000253	1.5%	1.40 [−0.56, 3.36]
SIL026	−0.5	0.699906	2.1%	−0.50 [−1.87, 0.87]
SIL027	0.92	2.077352	0.6%	0.92 [−3.15, 4.99]
SIL030	1.1	0.699719	2.1%	1.10 [−0.27, 2.47]
SIL034	2.66	1.069968	1.4%	2.66 [0.56, 4.76]
SIL15	0	2.18	0.5%	0.00 [−4.27, 4.27]
SIL16	−1.2	0.727055	2.0%	−1.20 [−2.63, 0.23]
SIL25	2.3	0.772015	1.9%	2.30 [0.79, 3.81]
SIL35	4.56	0.730352	2.0%	4.56 [3.13, 5.99]
SIL36	1.1	0.612152	2.3%	1.10 [−0.10, 2.30]
SIL40	1.38	0.741728	2.0%	1.38 [−0.07, 2.83]
SIL41b	2.9	0.683699	2.1%	2.90 [1.56, 4.24]
SIL44	1.7	1.270435	1.2%	1.70 [−0.79, 4.19]
SIL51	4.5	0.781353	1.9%	4.50 [2.97, 6.03]
SIL53	2.5	1.549244	0.9%	2.50 [−0.54, 5.54]
TAD005	0.87	0.771589	1.9%	0.87 [−0.64, 2.38]
TAD006	2.3	1.60068	0.9%	2.30 [−0.84, 5.44]
TAD009	1.3	0.612152	2.3%	1.30 [0.10, 2.50]
TAD01	−0.9	1.099852	1.4%	−0.90 [−3.06, 1.26]
TAD012	0.49	0.781353	1.9%	0.49 [−1.04, 2.02]
TAD015	3.3	0.730573	2.0%	3.30 [1.87, 4.73]
TAD018	1.8	0.51001	2.5%	1.80 [0.80, 2.80]
TAD03	0.1	0.783276	1.9%	0.10 [−1.44, 1.64]
TAD06	0.7	0.719907	2.0%	0.70 [−0.71, 2.11]
TAD07	−0.2	1.08075	1.4%	−0.20 [−2.32, 1.92]
TAD08	0.3	0.79963	1.9%	0.30 [−1.27, 1.87]
TAD09	0.4	0.942857	1.6%	0.40 [−1.45, 2.25]
TAD10	1.4	0.689143	2.1%	1.40 [0.05, 2.75]
TAD16	1.1	0.6153	2.2%	1.10 [−0.11, 2.31]
TAD18	1	0.899244	1.7%	1.00 [−0.76, 2.76]
VAR001	2.3	0.7475	2.0%	2.30 [0.83, 3.77]
VAR003	2.6	0.676015	2.1%	2.60 [1.28, 3.92]
VAR006	2.05	0.474335	2.5%	2.05 [1.12, 2.98]
VAR007	1.6	0.994066	1.5%	1.60 [−0.35, 3.55]
VAR015	3.63	4.850045	0.1%	3.63 [−5.88, 13.14]
VAR018	1.4	0.52307	2.4%	1.40 [0.37, 2.43]
VAR019	1.4	0.48541	2.5%	1.40 [0.45, 2.35]
VAR02	1.2	0.594552	2.3%	1.20 [0.03, 2.37]
VAR04	3.1	0.783276	1.9%	3.10 [1.56, 4.64]
VAR05	1.5	0.469631	2.5%	1.50 [0.58, 2.42]
VAR11	0.5	0.403791	2.7%	0.50 [−0.29, 1.29]
VAR12	1.3	0.432945	2.6%	1.30 [0.45, 2.15]
**Total (95% CI)**			**100.0%**	**1.67 [1.33, 2.01]**

Heterogeneity: $Tau^2 = 0.96$; $Chi^2 = 157.98$, df = 52 ($P < 0.00001$); $I^2 = 67\%$

Test for overall effect: $Z = 9.61$ ($P < 0.00001$)

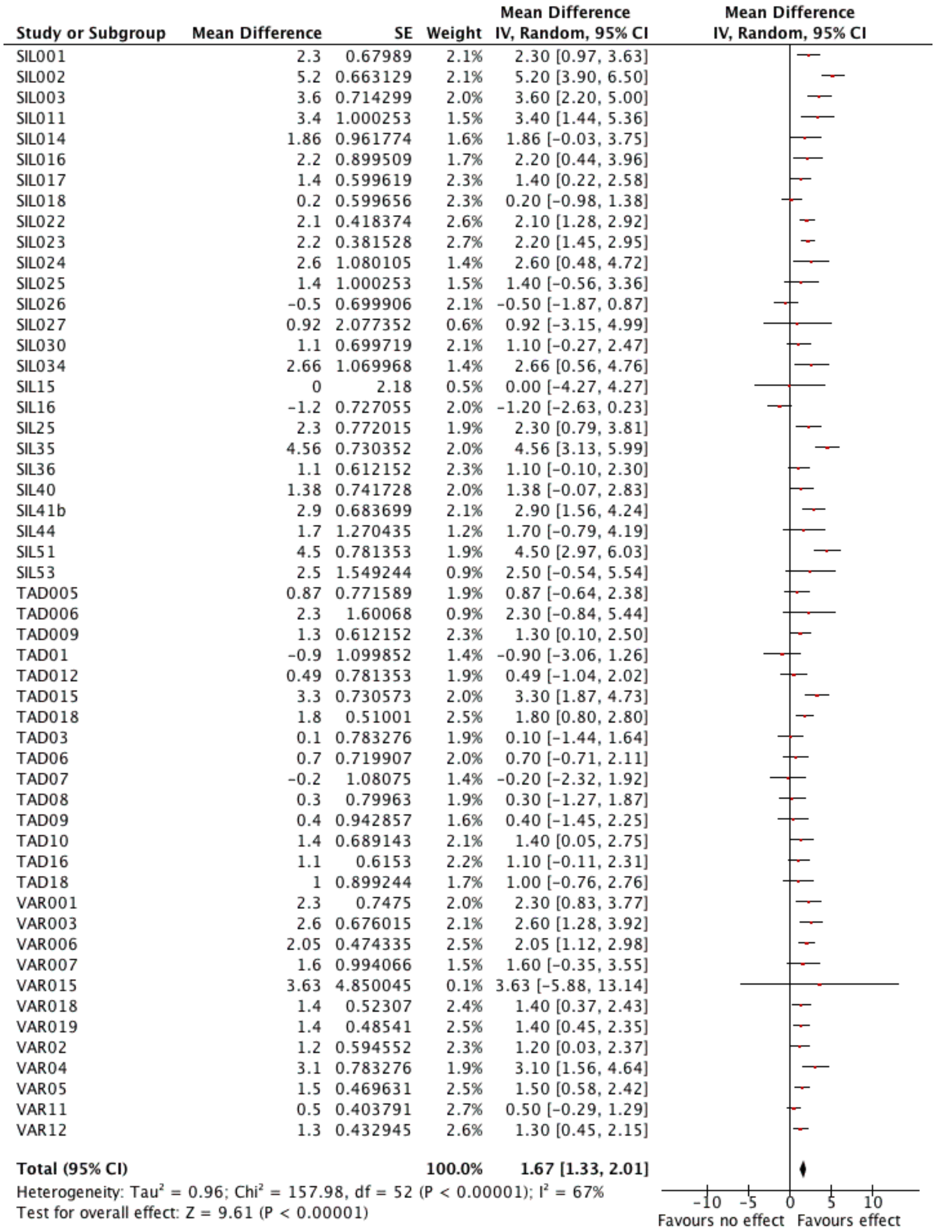

Forest plot of comparison: 7 IIEF-EF change scores: RCTs using parallel design, outcome: 7.1 Effect estimate placebo groups.

Figure 7 (Analysis 7.2)

Study or Subgroup	Mean Difference	SE	Weight	Mean Difference IV, Random, 95% CI
SIL001	9.1	0.679833	2.0%	9.10 [7.77, 10.43]
SIL002	11.7	0.663333	2.0%	11.70 [10.40, 13.00]
SIL003	9.3	0.714353	2.0%	9.30 [7.90, 10.70]
SIL011	8.9	1.000203	1.8%	8.90 [6.94, 10.86]
SIL014	9.17	0.979662	1.8%	9.17 [7.25, 11.09]
SIL016	8	0.899606	1.9%	8.00 [6.24, 9.76]
SIL017	8.7	0.599619	2.1%	8.70 [7.52, 9.88]
SIL018	6.1	0.600082	2.1%	6.10 [4.92, 7.28]
SIL022	9.5	0.316981	2.2%	9.50 [8.88, 10.12]
SIL023	3.6	0.390199	2.2%	3.60 [2.84, 4.36]
SIL024	7.2	0.750467	2.0%	7.20 [5.73, 8.67]
SIL025	6.8	1.20049	1.7%	6.80 [4.45, 9.15]
SIL026	2.1	0.599959	2.1%	2.10 [0.92, 3.28]
SIL027	5.79	1.54477	1.4%	5.79 [2.76, 8.82]
SIL030	5.4	0.599587	2.1%	5.40 [4.22, 6.58]
SIL034	10.37	0.920249	1.9%	10.37 [8.57, 12.17]
SIL15	7	1.158283	1.7%	7.00 [4.73, 9.27]
SIL16	8.4	0.714353	2.0%	8.40 [7.00, 9.80]
SIL25	12.3	0.743194	2.0%	12.30 [10.84, 13.76]
SIL35	10.66	0.720818	2.0%	10.66 [9.25, 12.07]
SIL36	10	0.716198	2.0%	10.00 [8.60, 11.40]
SIL40	5.98	0.73978	2.0%	5.98 [4.53, 7.43]
SIL41b	9.7	0.713379	2.0%	9.70 [8.30, 11.10]
SIL44	13.3	2.760809	0.8%	13.30 [7.89, 18.71]
SIL51	9.5	0.837855	1.9%	9.50 [7.86, 11.14]
SIL53	11.2	1.462624	1.5%	11.20 [8.33, 14.07]
TAD005	9.35	0.804142	1.9%	9.35 [7.77, 10.93]
TAD006	9.3	0.800167	1.9%	9.30 [7.73, 10.87]
TAD009	4.5	0.71988	2.0%	4.50 [3.09, 5.91]
TAD01	8	0.79949	1.9%	8.00 [6.43, 9.57]
TAD012	8.03	0.448769	2.1%	8.03 [7.15, 8.91]
TAD015	7.3	0.497391	2.1%	7.30 [6.33, 8.27]
TAD018	6.5	0.4899	2.1%	6.50 [5.54, 7.46]
TAD03	7.3	1.096016	1.7%	7.30 [5.15, 9.45]
TAD06	5.6	1.012517	1.8%	5.60 [3.62, 7.58]
TAD07	6.9	0.750126	2.0%	6.90 [5.43, 8.37]
TAD08	9.3	0.600161	2.1%	9.30 [8.12, 10.48]
TAD09	11.1	2.111129	1.1%	11.10 [6.96, 15.24]
TAD10	9.8	0.408084	2.1%	9.80 [9.00, 10.60]
TAD16	5.3	0.506429	2.1%	5.30 [4.31, 6.29]
TAD18	7.8	1.2	1.7%	7.80 [5.45, 10.15]
VAR001	8.8	0.436011	2.1%	8.80 [7.95, 9.65]
VAR003	9	0.386822	2.2%	9.00 [8.24, 9.76]
VAR006	7.79	0.634635	2.0%	7.79 [6.55, 9.03]
VAR007	10.9	0.819436	1.9%	10.90 [9.29, 12.51]
VAR015	4.56	4.589402	0.4%	4.56 [-4.44, 13.56]
VAR018	8.6	0.547024	2.1%	8.60 [7.53, 9.67]
VAR019	8.5	0.627571	2.0%	8.50 [7.27, 9.73]
VAR02	9	0.562143	2.1%	9.00 [7.90, 10.10]
VAR04	12.6	1.144751	1.7%	12.60 [10.36, 14.84]
VAR05	10.8	0.636719	2.0%	10.80 [9.55, 12.05]
VAR11	8.2	0.50824	2.1%	8.20 [7.20, 9.20]
VAR12	8.8	0.463406	2.1%	8.80 [7.89, 9.71]
**Total (95% CI)**			**100.0%**	**8.33 [7.73, 8.93]**

Heterogeneity: $Tau^2 = 4.17$; $Chi^2 = 574.81$, df = 52 (P < 0.00001); $I^2 = 91\%$
Test for overall effect: Z = 27.33 (P < 0.00001)

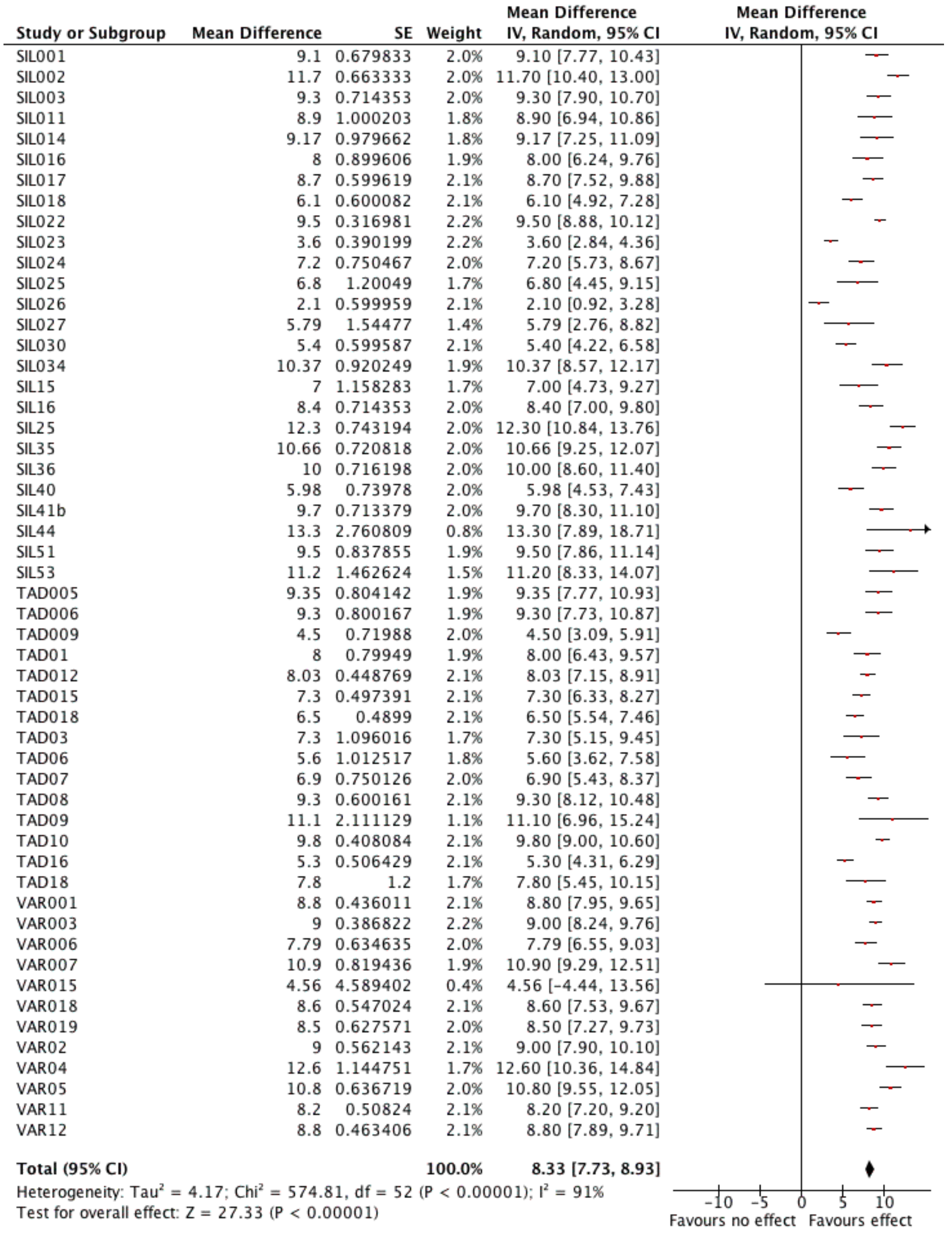

Forest plot of comparison: 7 IIEF-EF change scores: RCTs using parallel design, outcome: 7.2 Effect estimate intervention groups.

Figure 8 (Analysis 8.1)

Study or Subgroup	Mean Difference	SE	Weight	Mean Difference IV, Random, 95% CI
SIL006	6.38	0.966705	17.0%	6.38 [4.49, 8.27]
SIL007	0.3	1.753625	11.9%	0.30 [-3.14, 3.74]
SIL028	1.24	1.247726	15.1%	1.24 [-1.21, 3.69]
SIL1b	0.8	5.641379	2.4%	0.80 [-10.26, 11.86]
SIL2a	0.9	4.246676	3.8%	0.90 [-7.42, 9.22]
SIL2f	5.3	1.494917	13.5%	5.30 [2.37, 8.23]
VAR017	3	0.59794	19.3%	3.00 [1.83, 4.17]
VAR020	0.48	0.966705	17.0%	0.48 [-1.41, 2.37]
**Total (95% CI)**			**100.0%**	**2.74 [0.94, 4.54]**

Heterogeneity: $Tau^2 = 4.02$; $Chi^2 = 26.13$, df = 7 (P = 0.0005); $I^2 = 73\%$
Test for overall effect: Z = 2.98 (P = 0.003)

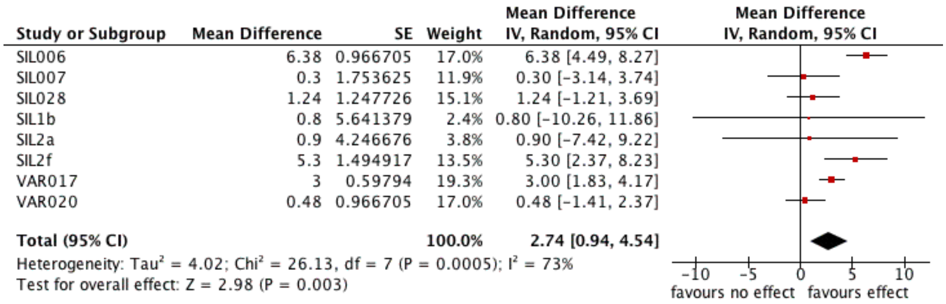

Forest plot of comparison: 8 IIEF-EF change scores: RCTs using crossover design, outcome: 8.1 Effect estimate placebo groups.

Figure 9 (Analysis 8.2)

Study or Subgroup	Mean Difference	SE	Weight	Mean Difference IV, Random, 95% CI
SIL006	11.08	1.358089	15.8%	11.08 [8.42, 13.74]
SIL007	9.3	1.753625	11.5%	9.30 [5.86, 12.74]
SIL028	6.39	1.187939	18.2%	6.39 [4.06, 8.72]
SIL1b	6	5.1878	1.8%	6.00 [-4.17, 16.17]
SIL2a	7.2	4.0184	3.0%	7.20 [-0.68, 15.08]
SIL2f	13.3	2.87	5.4%	13.30 [7.67, 18.93]
VAR017	10.1	0.5949	28.8%	10.10 [8.93, 11.27]
VAR020	8.95	1.390048	15.4%	8.95 [6.23, 11.67]
**Total (95% CI)**			**100.0%**	**9.32 [7.90, 10.74]**

Heterogeneity: $Tau^2 = 1.47$; $Chi^2 = 11.92$, df = 7 (P = 0.10); $I^2 = 41\%$
Test for overall effect: Z = 12.87 (P < 0.00001)

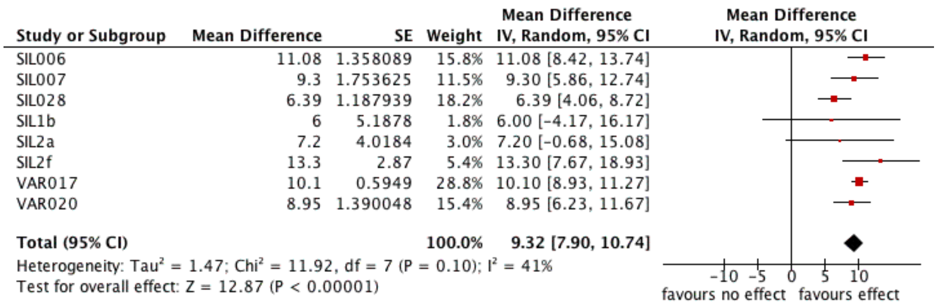

Forest plot of comparison: 8 IIEF-EF change scores: RCTs using crossover design, outcome: 8.2 Effect estimate intervention groups.

Figure 10 (Analysis 9.1)

Study or Subgroup	Mean Difference	SE	Weight	Mean Difference IV, Random, 95% CI
**9.1.1 Not commercial (Public / Mixed funding)**				
SIL027	0.92	2.0774	0.9%	0.92 [-3.15, 4.99]
SIL15	0	2.18	0.8%	0.00 [-4.27, 4.27]
SIL1b	0.8	1.31	1.7%	0.80 [-1.77, 3.37]
SIL2f	5.3	1.49	1.5%	5.30 [2.38, 8.22]
SIL44	1.7	1.27043518	1.8%	1.70 [-0.79, 4.19]
**Subtotal (95% CI)**			**6.7%**	**1.92 [0.08, 3.76]**
Heterogeneity: Tau² = 1.81; Chi² = 6.91, df = 4 (P = 0.14); I² = 42%				
Test for overall effect: Z = 2.05 (P = 0.04)				
**9.1.3 Commercial**				
SIL001	2.3	0.6798897	3.4%	2.30 [0.97, 3.63]
SIL002	5.2	0.66312884	3.4%	5.20 [3.90, 6.50]
SIL011	3.4	1.0002526	2.4%	3.40 [1.44, 5.36]
SIL014	1.86	0.9617741	2.5%	1.86 [-0.03, 3.75]
SIL016	2.2	0.89950884	2.7%	2.20 [0.44, 3.96]
SIL017	1.4	0.59961949	3.6%	1.40 [0.22, 2.58]
SIL018	0.2	0.59965555	3.6%	0.20 [-0.98, 1.38]
SIL022	2.1	0.41837395	4.3%	2.10 [1.28, 2.92]
SIL025	1.4	1.0002526	2.4%	1.40 [-0.56, 3.36]
SIL026	-0.5	0.69990606	3.3%	-0.50 [-1.87, 0.87]
SIL030	1.1	0.69971892	3.3%	1.10 [-0.27, 2.47]
SIL034	2.66	1.06996831	2.2%	2.66 [0.56, 4.76]
TAD005	0.87	0.77158927	3.1%	0.87 [-0.64, 2.38]
TAD006	2.3	1.60067954	1.3%	2.30 [-0.84, 5.44]
TAD01	-0.9	1.09985226	2.2%	-0.90 [-3.06, 1.26]
TAD012	0.49	0.78135299	3.0%	0.49 [-1.04, 2.02]
TAD015	3.3	0.73057328	3.2%	3.30 [1.87, 4.73]
TAD018	1.8	0.5100098	4.0%	1.80 [0.80, 2.80]
TAD03	0.1	0.78327589	3.0%	0.10 [-1.44, 1.64]
TAD07	-0.2	1.08074954	2.2%	-0.20 [-2.32, 1.92]
TAD08	0.3	0.79963012	3.0%	0.30 [-1.27, 1.87]
TAD09	0.4	0.94285714	2.6%	0.40 [-1.45, 2.25]
TAD10	1.4	0.68914269	3.3%	1.40 [0.05, 2.75]
TAD16	1.1	0.61529978	3.6%	1.10 [-0.11, 2.31]
TAD18	1	0.89924413	2.7%	1.00 [-0.76, 2.76]
VAR006	2.05	0.47433528	4.1%	2.05 [1.12, 2.98]
VAR007	1.6	0.99406573	2.4%	1.60 [-0.35, 3.55]
VAR015	3.63	4.85004527	0.2%	3.63 [-5.88, 13.14]
VAR018	1.4	0.5230701	3.9%	1.40 [0.37, 2.43]
VAR019	1.4	0.48540962	4.1%	1.40 [0.45, 2.35]
VAR11	0.5	0.40379108	4.3%	0.50 [-0.29, 1.29]
**Subtotal (95% CI)**			**93.3%**	**1.42 [0.99, 1.85]**
Heterogeneity: Tau² = 0.87; Chi² = 84.65, df = 30 (P < 0.00001); I² = 65%				
Test for overall effect: Z = 6.48 (P < 0.00001)				
**Total (95% CI)**			**100.0%**	**1.46 [1.04, 1.87]**
Heterogeneity: Tau² = 0.88; Chi² = 92.12, df = 35 (P < 0.00001); I² = 62%				
Test for overall effect: Z = 6.85 (P < 0.00001)				
Test for subgroup differences: Chi² = 0.27, df = 1 (P = 0.60), I² = 0%				

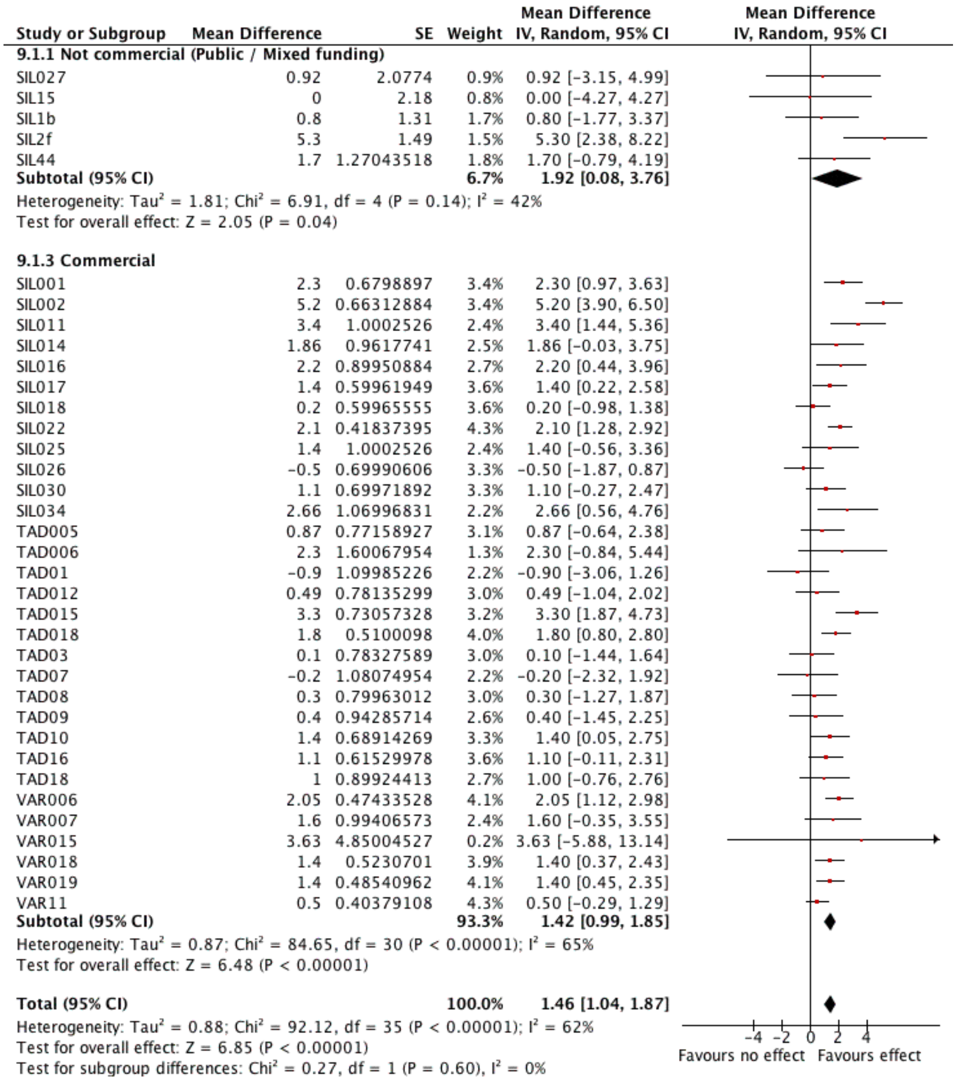

Forest plot of comparison: 9 IIEF-EF change scores: Commercial Funding, outcome: 9.1 Effect estimate placebo groups.

Figure 11 (Analysis 9.2)

Study or Subgroup	Mean Difference	SE	Weight	Mean Difference IV, Random, 95% CI
**9.2.1 Not commercial (Public / Mixed funding)**				
SIL027	5.79	1.54	2.1%	5.79 [2.77, 8.81]
SIL15	7	1.91	1.7%	7.00 [3.26, 10.74]
SIL1b	6	2.43	1.3%	6.00 [1.24, 10.76]
SIL2f	13.3	2.87	1.1%	13.30 [7.67, 18.93]
SIL44	13.3	2.76	1.1%	13.30 [7.89, 18.71]
**Subtotal (95% CI)**			**7.3%**	**8.56 [5.47, 11.64]**
Heterogeneity: Tau² = 7.25; Chi² = 10.07, df = 4 (P = 0.04); I² = 60%				
Test for overall effect: Z = 5.44 (P < 0.00001)				
**9.2.3 Commercial**				
SIL001	9.1	0.68	3.0%	9.10 [7.77, 10.43]
SIL002	11.7	0.66	3.1%	11.70 [10.41, 12.99]
SIL011	8.9	1	2.7%	8.90 [6.94, 10.86]
SIL014	9.17	0.98	2.7%	9.17 [7.25, 11.09]
SIL016	8	0.9	2.8%	8.00 [6.24, 9.76]
SIL017	8.7	0.6	3.1%	8.70 [7.52, 9.88]
SIL018	6.1	0.6	3.1%	6.10 [4.92, 7.28]
SIL022	9.5	0.32	3.3%	9.50 [8.87, 10.13]
SIL025	6.8	1.2	2.5%	6.80 [4.45, 9.15]
SIL026	2.1	0.6	3.1%	2.10 [0.92, 3.28]
SIL030	5.4	0.6	3.1%	5.40 [4.22, 6.58]
SIL034	10.37	0.92	2.8%	10.37 [8.57, 12.17]
TAD005	9.35	0.8	2.9%	9.35 [7.78, 10.92]
TAD006	9.3	0.8	2.9%	9.30 [7.73, 10.87]
TAD01	8	0.8	2.9%	8.00 [6.43, 9.57]
TAD012	8.03	0.45	3.2%	8.03 [7.15, 8.91]
TAD015	7.3	0.5	3.2%	7.30 [6.32, 8.28]
TAD018	6.5	0.49	3.2%	6.50 [5.54, 7.46]
TAD03	7.3	1.1	2.6%	7.30 [5.14, 9.46]
TAD07	6.9	0.75	3.0%	6.90 [5.43, 8.37]
TAD08	9.3	0.6	3.1%	9.30 [8.12, 10.48]
TAD09	11.1	0.56	3.2%	11.10 [10.00, 12.20]
TAD10	9.8	0.41	3.3%	9.80 [9.00, 10.60]
TAD16	5.3	0.51	3.2%	5.30 [4.30, 6.30]
TAD18	7.8	1.2	2.5%	7.80 [5.45, 10.15]
VAR006	7.79	0.63	3.1%	7.79 [6.56, 9.02]
VAR007	10.9	0.82	2.9%	10.90 [9.29, 12.51]
VAR015	4.56	0.96	2.7%	4.56 [2.68, 6.44]
VAR018	8.6	0.55	3.2%	8.60 [7.52, 9.68]
VAR019	8.5	0.63	3.1%	8.50 [7.27, 9.73]
VAR11	8.2	0.51	3.2%	8.20 [7.20, 9.20]
**Subtotal (95% CI)**			**92.7%**	**8.08 [7.35, 8.80]**
Heterogeneity: Tau² = 3.71; Chi² = 320.13, df = 30 (P < 0.00001); I² = 91%				
Test for overall effect: Z = 21.84 (P < 0.00001)				
**Total (95% CI)**			**100.0%**	**8.10 [7.40, 8.80]**
Heterogeneity: Tau² = 3.72; Chi² = 330.34, df = 35 (P < 0.00001); I² = 89%				
Test for overall effect: Z = 22.71 (P < 0.00001)				
Test for subgroup differences: Chi² = 0.09, df = 1 (P = 0.77), I² = 0%				

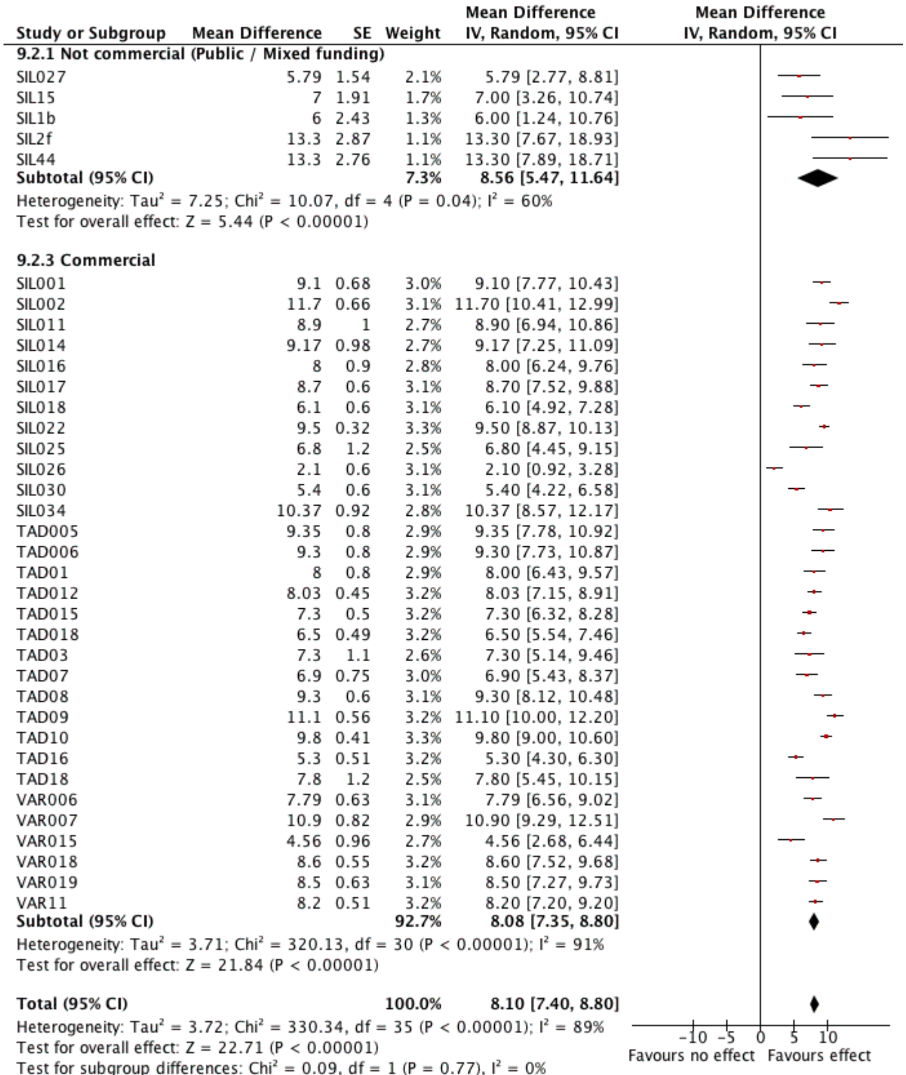

Forest plot of comparison: 9 IIEF-EF change scores: Commercial Funding, outcome: 9.2 Effect estimate intervention groups.

Figure 12 (Analysis 10.1)

Study or Subgroup	Mean Difference	SE	Weight	Mean Difference IV, Random, 95% CI
**10.1.1 Across continents**				
SIL001	2.3	0.6798897	2.5%	2.30 [0.97, 3.63]
SIL002	5.2	0.66312884	2.6%	5.20 [3.90, 6.50]
SIL017	1.4	0.59961949	2.7%	1.40 [0.22, 2.58]
SIL022	2.1	0.41837395	3.2%	2.10 [1.28, 2.92]
SIL48	2.5	0.96888889	1.9%	2.50 [0.60, 4.40]
TAD005	0.87	0.77158927	2.3%	0.87 [−0.64, 2.38]
TAD006	2.3	1.60067954	1.0%	2.30 [−0.84, 5.44]
TAD009	1.3	0.61215244	2.7%	1.30 [0.10, 2.50]
TAD012	0.49	0.78135299	2.3%	0.49 [−1.04, 2.02]
TAD018	1.8	0.5100098	3.0%	1.80 [0.80, 2.80]
TAD06	0.7	1.04976464	1.7%	0.70 [−1.36, 2.76]
TAD10	1.4	0.68914269	2.5%	1.40 [0.05, 2.75]
TAD16	1.1	0.61529978	2.7%	1.10 [−0.11, 2.31]
VAR004	1.6	0.52840716	2.9%	1.60 [0.56, 2.64]
VAR005	2	0.38590553	3.3%	2.00 [1.24, 2.76]
VAR018	1.4	0.5230701	2.9%	1.40 [0.37, 2.43]
VAR019	1.4	0.48540962	3.0%	1.40 [0.45, 2.35]
VAR05	1.5	0.46963101	3.1%	1.50 [0.58, 2.42]
VAR07	0.8	0.46971163	3.1%	0.80 [−0.12, 1.72]
**Subtotal (95% CI)**			**49.2%**	**1.68 [1.27, 2.09]**
Heterogeneity: $Tau^2 = 0.45$; $Chi^2 = 41.87$, df = 18 (P = 0.001); $I^2 = 57\%$				
Test for overall effect: Z = 7.96 (P < 0.00001)				
**10.1.2 Africa**				
SIL47	3.4	0.69350381	2.5%	3.40 [2.04, 4.76]
**Subtotal (95% CI)**			**2.5%**	**3.40 [2.04, 4.76]**
Heterogeneity: Not applicable				
Test for overall effect: Z = 4.90 (P < 0.00001)				
**10.1.3 Asia**				
SIL006	6.38	0.95143	1.9%	6.38 [4.52, 8.24]
SIL007	0.3	1.75362482	0.9%	0.30 [−3.14, 3.74]
SIL009	4.6	1.41138514	1.2%	4.60 [1.83, 7.37]
SIL023	2.2	0.38152839	3.3%	2.20 [1.45, 2.95]
SIL025	1.4	1.0002526	1.8%	1.40 [−0.56, 3.36]
SIL027	0.92	2.07735223	0.7%	0.92 [−3.15, 4.99]
**Subtotal (95% CI)**			**9.8%**	**2.84 [1.04, 4.64]**
Heterogeneity: $Tau^2 = 3.49$; $Chi^2 = 22.70$, df = 5 (P = 0.0004); $I^2 = 78\%$				
Test for overall effect: Z = 3.09 (P = 0.002)				
**10.1.4 Europe**				
SIL13	0.1	0.71429868	2.4%	0.10 [−1.30, 1.50]
SIL36	1.1	0.61215244	2.7%	1.10 [−0.10, 2.30]
SIL37	1.53	0.68587126	2.5%	1.53 [0.19, 2.87]
TAD015	3.3	0.73057328	2.4%	3.30 [1.87, 4.73]
TAD03	0.1	0.78327589	2.3%	0.10 [−1.44, 1.64]
TAD09	0.4	0.94285714	1.9%	0.40 [−1.45, 2.25]
VAR006	2.05	0.47433528	3.0%	2.05 [1.12, 2.98]
VAR007	1.6	0.99406573	1.8%	1.60 [−0.35, 3.55]
VAR015	3.63	4.85004527	0.1%	3.63 [−5.88, 13.14]
**Subtotal (95% CI)**			**19.3%**	**1.34 [0.61, 2.07]**
Heterogeneity: $Tau^2 = 0.58$; $Chi^2 = 16.35$, df = 8 (P = 0.04); $I^2 = 51\%$				
Test for overall effect: Z = 3.61 (P = 0.0003)				
**10.1.5 North America**				
SIL003	3.6	0.71429868	2.4%	3.60 [2.20, 5.00]
SIL011	3.4	1.0002526	1.8%	3.40 [1.44, 5.36]
SIL014	1.86	0.9617741	1.9%	1.86 [−0.03, 3.75]
SIL026	−0.5	0.69990606	2.5%	−0.50 [−1.87, 0.87]
SIL035a	0.2	0		Not estimable
SIL035b	−0.9	0		Not estimable
SIL31	0.2	0.6729535	2.5%	0.20 [−1.12, 1.52]
TAD01	−0.9	1.09985226	1.6%	−0.90 [−3.06, 1.26]
TAD07	−0.2	1.08074954	1.7%	−0.20 [−2.32, 1.92]
TAD08	0.3	0.79963012	2.2%	0.30 [−1.27, 1.87]
**Subtotal (95% CI)**			**16.8%**	**0.99 [−0.25, 2.22]**
Heterogeneity: $Tau^2 = 2.40$; $Chi^2 = 30.79$, df = 7 (P < 0.0001); $I^2 = 77\%$				
Test for overall effect: Z = 1.56 (P = 0.12)				
**10.1.6 South America**				
SIL53	2.5	1.54924372	1.1%	2.50 [−0.54, 5.54]
**Subtotal (95% CI)**			**1.1%**	**2.50 [−0.54, 5.54]**
Heterogeneity: Not applicable				
Test for overall effect: Z = 1.61 (P = 0.11)				
**10.1.7 Oceania**				
SIL028	1.24	1.24772593	1.4%	1.24 [−1.21, 3.69]
**Subtotal (95% CI)**			**1.4%**	**1.24 [−1.21, 3.69]**
Heterogeneity: Not applicable				
Test for overall effect: Z = 0.99 (P = 0.32)				
**Total (95% CI)**			**100.0%**	**1.66 [1.29, 2.03]**
Heterogeneity: $Tau^2 = 0.94$; $Chi^2 = 133.50$, df = 44 (P < 0.00001); $I^2 = 67\%$				
Test for overall effect: Z = 8.84 (P < 0.00001)				
Test for subgroup differences: $Chi^2 = 10.17$, df = 6 (P = 0.12), $I^2 = 41.0\%$				

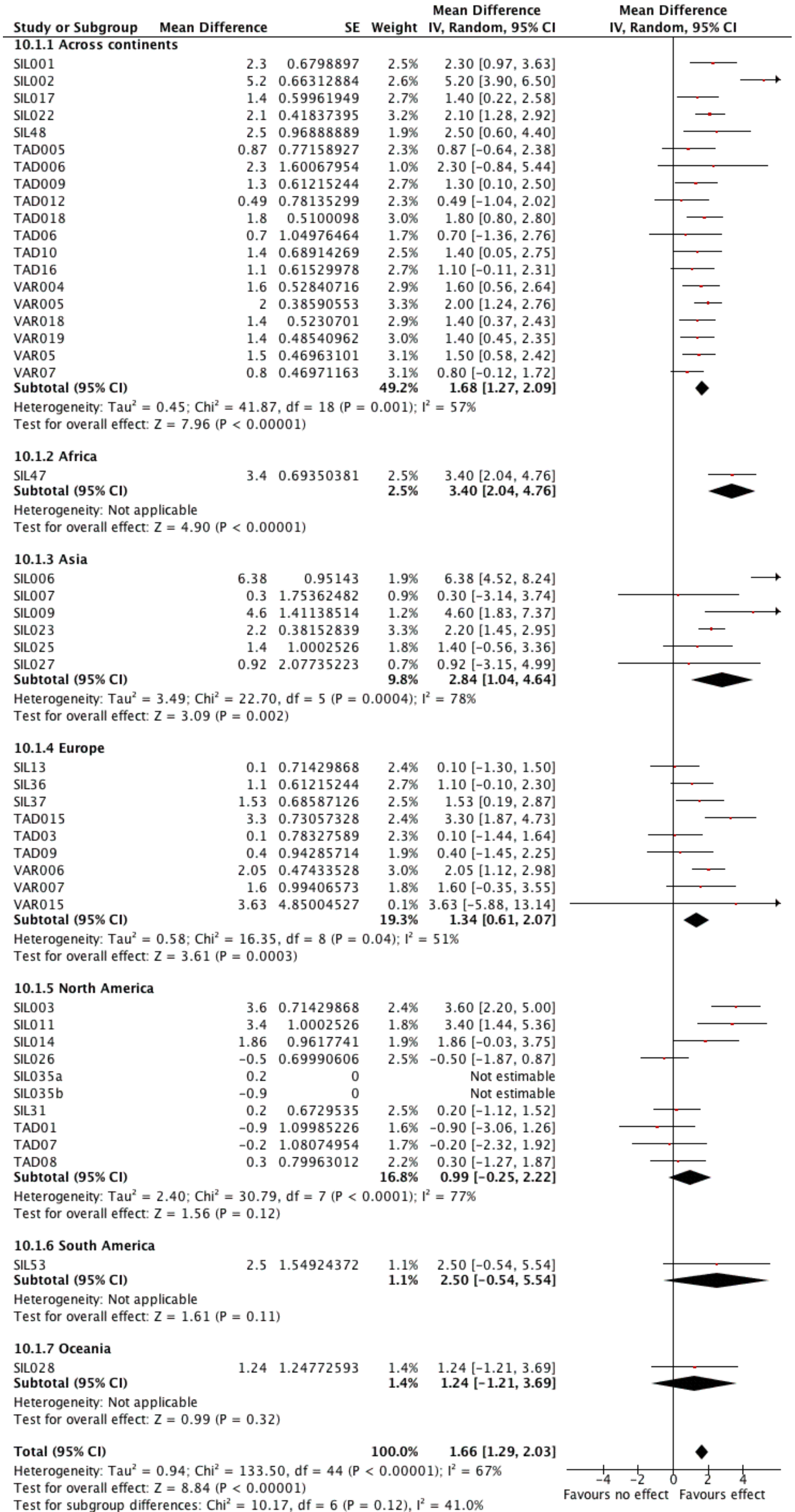

Forest plot of comparison: 10 IIEF-EF change scores: Continent, outcome: 10.1 Effect estimate placebo groups.

Figure 13 (Analysis 10.2)

Study or Subgroup	Mean Difference	SE	Weight	Mean Difference IV, Random, 95% CI
**10.2.1 Across continents**				
SIL001	9.1	0.67983266	2.3%	9.10 [7.77, 10.43]
SIL002	11.7	0.66333333	2.3%	11.70 [10.40, 13.00]
SIL017	8.7	0.59961949	2.3%	8.70 [7.52, 9.88]
SIL022	9.5	0.31698117	2.4%	9.50 [8.88, 10.12]
SIL48	7	0.95863335	2.1%	7.00 [5.12, 8.88]
TAD005	9.35	0.80414166	2.2%	9.35 [7.77, 10.93]
TAD006	9.3	0.80016665	2.2%	9.30 [7.73, 10.87]
TAD009	4.5	0.71988043	2.2%	4.50 [3.09, 5.91]
TAD012	8.03	0.44876926	2.4%	8.03 [7.15, 8.91]
TAD018	6.5	0.48989996	2.4%	6.50 [5.54, 7.46]
TAD06	5.6	1.03344151	2.0%	5.60 [3.57, 7.63]
TAD10	9.8	0.40808442	2.4%	9.80 [9.00, 10.60]
TAD16	5.3	0.50642857	2.4%	5.30 [4.31, 6.29]
VAR004	9.5	0.51042815	2.4%	9.50 [8.50, 10.50]
VAR005	10	0.39467611	2.4%	10.00 [9.23, 10.77]
VAR018	8.6	0.54702374	2.3%	8.60 [7.53, 9.67]
VAR019	8.5	0.62757064	2.3%	8.50 [7.27, 9.73]
VAR05	10.8	0.63671908	2.3%	10.80 [9.55, 12.05]
VAR07	8.3	0.46226179	2.4%	8.30 [7.39, 9.21]
**Subtotal (95% CI)**			**43.5%**	**8.47 [7.70, 9.24]**
Heterogeneity: $Tau^2 = 2.53$; $Chi^2 = 174.37$, df = 18 (P < 0.00001); $I^2 = 90\%$				
Test for overall effect: Z = 21.60 (P < 0.00001)				
**10.2.2 Africa**				
SIL47	11.2	0.67349833	2.3%	11.20 [9.88, 12.52]
**Subtotal (95% CI)**			**2.3%**	**11.20 [9.88, 12.52]**
Heterogeneity: Not applicable				
Test for overall effect: Z = 16.63 (P < 0.00001)				
**10.2.3 Asia**				
SIL006	11.08	1.49240501	1.7%	11.08 [8.15, 14.01]
SIL007	9.3	1.75362482	1.5%	9.30 [5.86, 12.74]
SIL009	8.8	0.90647888	2.1%	8.80 [7.02, 10.58]
SIL023	3.6	0.39019949	2.4%	3.60 [2.84, 4.36]
SIL025	6.8	1.2004901	1.9%	6.80 [4.45, 9.15]
SIL027	5.79	1.54476998	1.6%	5.79 [2.76, 8.82]
**Subtotal (95% CI)**			**11.2%**	**7.44 [4.69, 10.18]**
Heterogeneity: $Tau^2 = 10.17$; $Chi^2 = 55.43$, df = 5 (P < 0.00001); $I^2 = 91\%$				
Test for overall effect: Z = 5.31 (P < 0.00001)				
**10.2.4 Europe**				
SIL13	9.5	0.68445024	2.3%	9.50 [8.16, 10.84]
SIL36	10	0.71619815	2.2%	10.00 [8.60, 11.40]
SIL37	10.85	0.66333333	2.3%	10.85 [9.55, 12.15]
TAD015	7.3	0.49739114	2.4%	7.30 [6.33, 8.27]
TAD03	7.3	1.09601551	2.0%	7.30 [5.15, 9.45]
TAD09	11.1	2.11112943	1.3%	11.10 [6.96, 15.24]
VAR006	7.79	0.63463489	2.3%	7.79 [6.55, 9.03]
VAR007	10.9	0.81943603	2.2%	10.90 [9.29, 12.51]
VAR015	4.56	4.58940225	0.4%	4.56 [−4.44, 13.56]
**Subtotal (95% CI)**			**17.3%**	**9.14 [7.98, 10.30]**
Heterogeneity: $Tau^2 = 2.10$; $Chi^2 = 35.22$, df = 8 (P < 0.0001); $I^2 = 77\%$				
Test for overall effect: Z = 15.43 (P < 0.00001)				
**10.2.5 North America**				
SIL003	9.3	0.71435285	2.2%	9.30 [7.90, 10.70]
SIL011	8.9	1.00020314	2.0%	8.90 [6.94, 10.86]
SIL014	9.17	0.97966168	2.1%	9.17 [7.25, 11.09]
SIL026	2.1	0.5999588	2.3%	2.10 [0.92, 3.28]
SIL035a	6.1	0.56424471	2.3%	6.10 [4.99, 7.21]
SIL035b	6.8	0.81482317	2.2%	6.80 [5.20, 8.40]
SIL31	11.1	0.68256437	2.3%	11.10 [9.76, 12.44]
TAD01	8	0.79948976	2.2%	8.00 [6.43, 9.57]
TAD07	6.9	0.75012602	2.2%	6.90 [5.43, 8.37]
TAD08	9.3	0.60016134	2.3%	9.30 [8.12, 10.48]
**Subtotal (95% CI)**			**22.1%**	**7.74 [6.00, 9.49]**
Heterogeneity: $Tau^2 = 7.32$; $Chi^2 = 136.87$, df = 9 (P < 0.00001); $I^2 = 93\%$				
Test for overall effect: Z = 8.71 (P < 0.00001)				
**10.2.6 South America**				
SIL53	11.2	1.46262421	1.7%	11.20 [8.33, 14.07]
**Subtotal (95% CI)**			**1.7%**	**11.20 [8.33, 14.07]**
Heterogeneity: Not applicable				
Test for overall effect: Z = 7.66 (P < 0.00001)				
**10.2.7 Oceania**				
SIL028	6.39	1.18793939	1.9%	6.39 [4.06, 8.72]
**Subtotal (95% CI)**			**1.9%**	**6.39 [4.06, 8.72]**
Heterogeneity: Not applicable				
Test for overall effect: Z = 5.38 (P < 0.00001)				
**Total (95% CI)**			**100.0%**	**8.35 [7.69, 9.02]**
Heterogeneity: $Tau^2 = 4.60$; $Chi^2 = 555.98$, df = 46 (P < 0.00001); $I^2 = 92\%$				
Test for overall effect: Z = 24.68 (P < 0.00001)				
Test for subgroup differences: $Chi^2 = 22.75$, df = 6 (P = 0.0009), $I^2 = 73.6\%$				

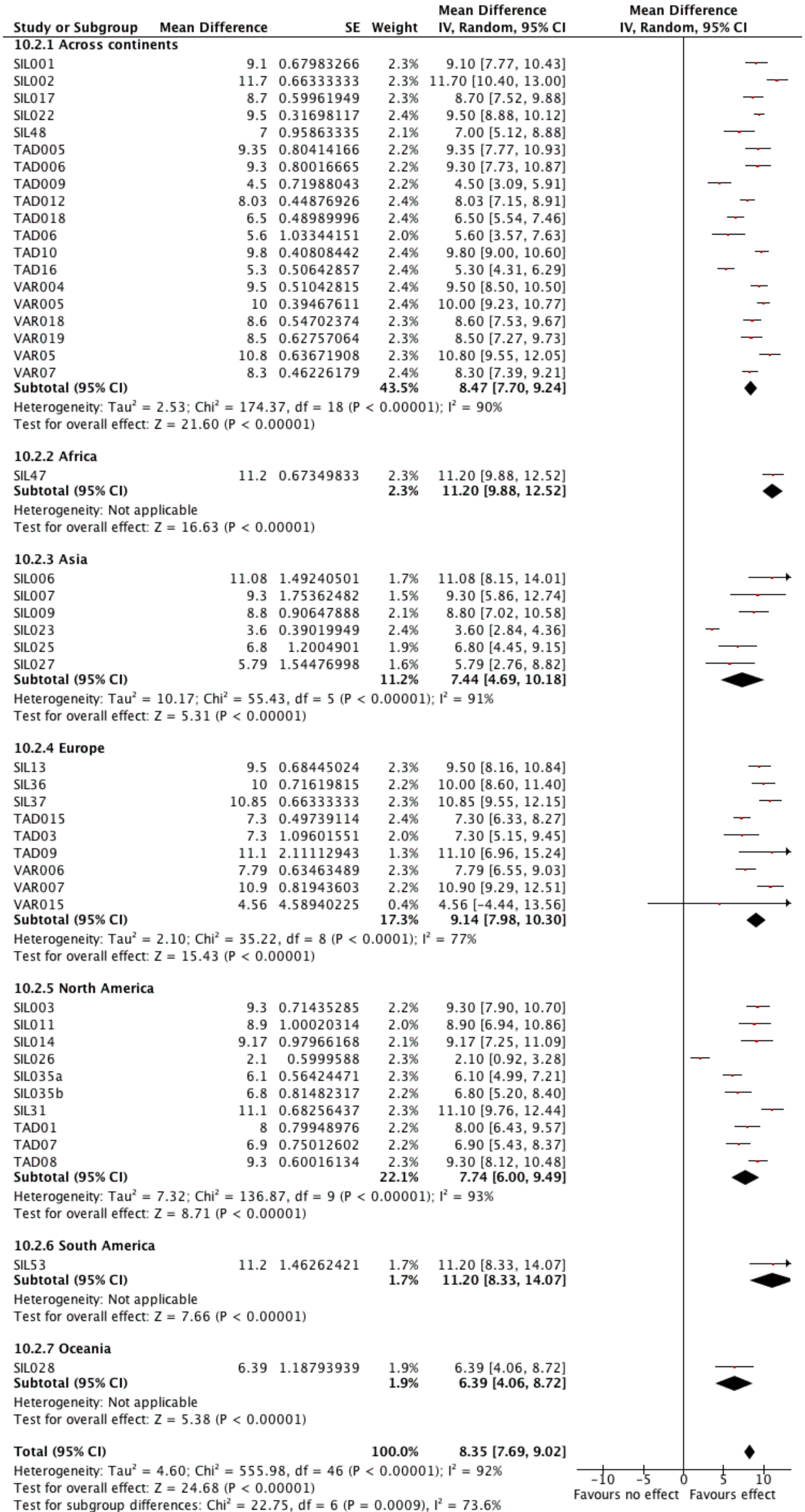

Forest plot of comparison: 10 IIEF-EF change scores: Continent, outcome: 10.2 Effect estimate intervention groups.

Figure 14 (Analysis 11.1)

Study or Subgroup	Mean Difference	SE	Weight	Mean Difference IV, Random, 95% CI
**11.1.1 Singlecenter**				
SIL006	6.38	0.95143	1.6%	6.38 [4.52, 8.24]
SIL007	0.3	1.75362482	0.7%	0.30 [−3.14, 3.74]
SIL025	1.4	1.0002526	1.5%	1.40 [−0.56, 3.36]
SIL027	0.92	2.07735223	0.6%	0.92 [−3.15, 4.99]
SIL1b	0.8	5.64137864	0.1%	0.80 [−10.26, 11.86]
SIL44	1.7	1.27043518	1.2%	1.70 [−0.79, 4.19]
VAR015	3.63	4.85004527	0.1%	3.63 [−5.88, 13.14]
**Subtotal (95% CI)**			**5.8%**	**2.36 [0.08, 4.65]**
Heterogeneity: Tau² = 5.44; Chi² = 19.77, df = 6 (P = 0.003); I² = 70%				
Test for overall effect: Z = 2.03 (P = 0.04)				
**11.1.2 Multicenter**				
SIL001	2.3	0.6798897	2.1%	2.30 [0.97, 3.63]
SIL002	5.2	0.66312884	2.2%	5.20 [3.90, 6.50]
SIL003	3.6	0.71429868	2.1%	3.60 [2.20, 5.00]
SIL009	4.6	1.41138514	1.0%	4.60 [1.83, 7.37]
SIL011	3.4	1.0002526	1.5%	3.40 [1.44, 5.36]
SIL014	1.86	0.9617741	1.6%	1.86 [−0.03, 3.75]
SIL016	2.2	0.89950884	1.7%	2.20 [0.44, 3.96]
SIL017	1.4	0.59961949	2.3%	1.40 [0.22, 2.58]
SIL018	0.2	0.59965555	2.3%	0.20 [−0.98, 1.38]
SIL022	2.1	0.41837395	2.7%	2.10 [1.28, 2.92]
SIL023	2.2	0.38152839	2.8%	2.20 [1.45, 2.95]
SIL026	−0.5	0.69990606	2.1%	−0.50 [−1.87, 0.87]
SIL028	1.24	1.24772593	1.2%	1.24 [−1.21, 3.69]
SIL030	1.1	0.69971892	2.1%	1.10 [−0.27, 2.47]
SIL034	2.66	1.06996831	1.4%	2.66 [0.56, 4.76]
SIL035a	0.2	0.6208823	2.3%	0.20 [−1.02, 1.42]
SIL035b	−0.9	0.81363636	1.9%	−0.90 [−2.49, 0.69]
SIL53	2.5	1.54924372	0.9%	2.50 [−0.54, 5.54]
TAD001	0.1	1.3618352	1.1%	0.10 [−2.57, 2.77]
TAD004	2.1	0.94030178	1.6%	2.10 [0.26, 3.94]
TAD005	0.87	0.77158927	2.0%	0.87 [−0.64, 2.38]
TAD006	2.3	1.60067954	0.8%	2.30 [−0.84, 5.44]
TAD007	2.1	0.95714435	1.6%	2.10 [0.22, 3.98]
TAD008	1.2	1.07428196	1.4%	1.20 [−0.91, 3.31]
TAD009	1.3	0.88085302	1.7%	1.30 [−0.43, 3.03]
TAD01	−0.9	1.09985226	1.4%	−0.90 [−3.06, 1.26]
TAD012	0.49	0.78135299	1.9%	0.49 [−1.04, 2.02]
TAD015	3.3	0.73057328	2.0%	3.30 [1.87, 4.73]
TAD018	1.8	0.5100098	2.5%	1.80 [0.80, 2.80]
TAD02	1.1	0.76051316	2.0%	1.10 [−0.39, 2.59]
TAD04	2.6	0.95506746	1.6%	2.60 [0.73, 4.47]
TAD06	0.7	0.7199074	2.1%	0.70 [−0.71, 2.11]
TAD07	−0.2	1.08074954	1.4%	−0.20 [−2.32, 1.92]
TAD08	0.3	0.79963012	1.9%	0.30 [−1.27, 1.87]
TAD09	0.4	0.94285714	1.6%	0.40 [−1.45, 2.25]
TAD10	1.4	0.68914269	2.1%	1.40 [0.05, 2.75]
TAD11	2.4	0.69350381	2.1%	2.40 [1.04, 3.76]
TAD16	1.1	0.61529978	2.3%	1.10 [−0.11, 2.31]
TAD18	1	0.89924413	1.7%	1.00 [−0.76, 2.76]
VAR005	2	0.38590553	2.8%	2.00 [1.24, 2.76]
VAR006	2.05	0.47433528	2.6%	2.05 [1.12, 2.98]
VAR007	1.6	0.99406573	1.5%	1.60 [−0.35, 3.55]
VAR008	1.69	0.7199074	2.1%	1.69 [0.28, 3.10]
VAR018	1.4	0.5230701	2.5%	1.40 [0.37, 2.43]
VAR019	1.4	0.48540962	2.6%	1.40 [0.45, 2.35]
VAR020	0.48	0.96670525	1.6%	0.48 [−1.41, 2.37]
VAR09	1.6	0.64250837	2.2%	1.60 [0.34, 2.86]
VAR10	1.4	0.66312884	2.2%	1.40 [0.10, 2.70]
VAR11	0.5	0.40379108	2.8%	0.50 [−0.29, 1.29]
**Subtotal (95% CI)**			**94.2%**	**1.52 [1.19, 1.84]**
Heterogeneity: Tau² = 0.73; Chi² = 121.00, df = 48 (P < 0.00001); I² = 60%				
Test for overall effect: Z = 9.20 (P < 0.00001)				
**Total (95% CI)**			**100.0%**	**1.59 [1.25, 1.92]**
Heterogeneity: Tau² = 0.89; Chi² = 147.36, df = 55 (P < 0.00001); I² = 63%				
Test for overall effect: Z = 9.31 (P < 0.00001)				
Test for subgroup differences: Chi² = 0.52, df = 1 (P = 0.47), I² = 0%				

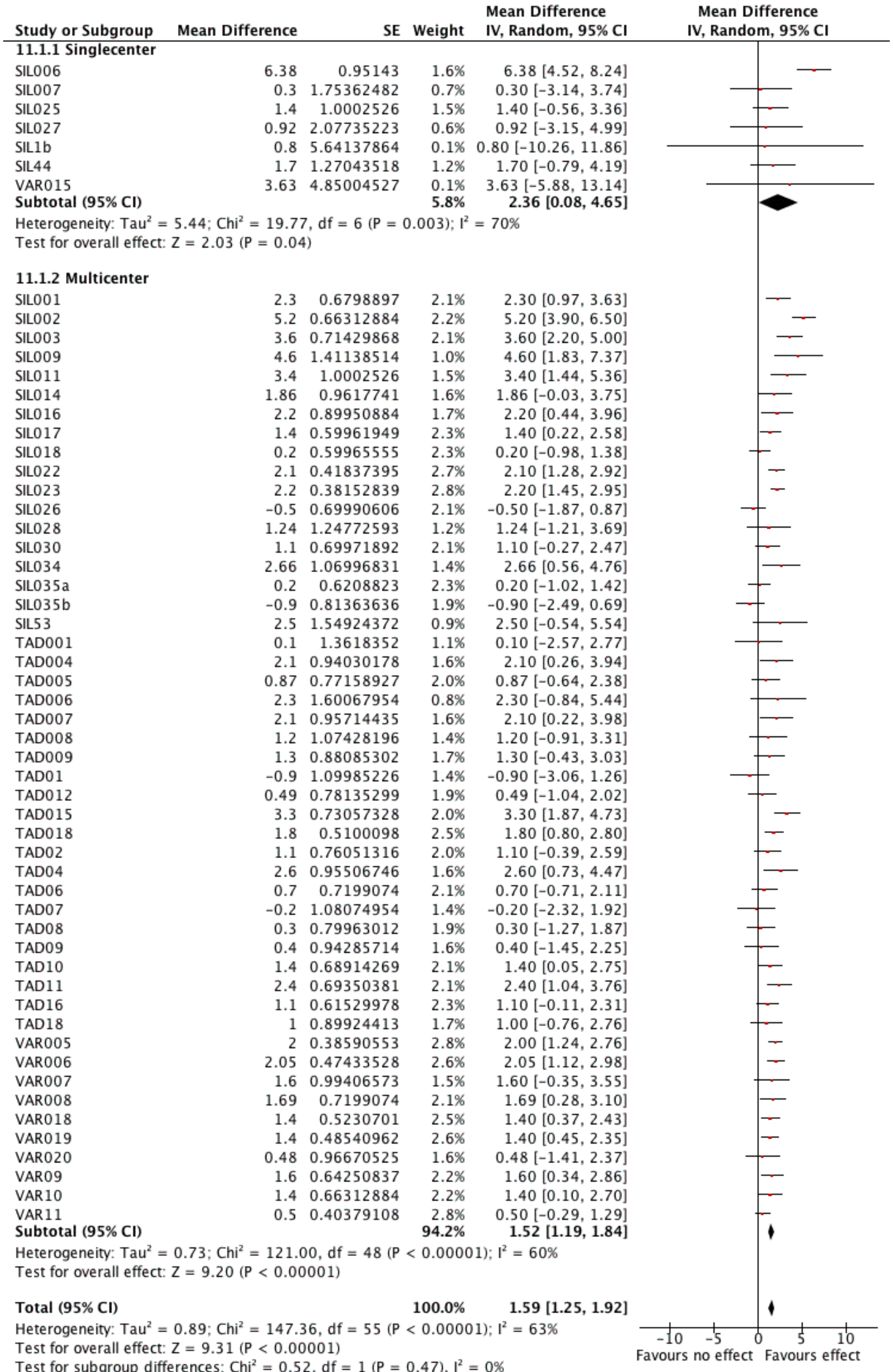

Forest plot of comparison: 11 IIEF-EF change scores: Single-center study, outcome: 11.1 Effect estimate placebo groups.

Figure 15 (Analysis 11.2)

Study or Subgroup	Mean Difference	SE	Weight	Mean Difference IV, Random, 95% CI
**11.2.1 Singlecenter**				
SIL006	11.08	1.49240501	1.5%	11.08 [8.15, 14.01]
SIL007	9.3	1.75362482	1.3%	9.30 [5.86, 12.74]
SIL025	6.8	1.2004901	1.6%	6.80 [4.45, 9.15]
SIL027	5.79	1.54476998	1.4%	5.79 [2.76, 8.82]
SIL1b	6	5.18780675	0.3%	6.00 [-4.17, 16.17]
SIL44	13.3	2.76080863	0.9%	13.30 [7.89, 18.71]
VAR015	4.56	4.58940225	0.4%	4.56 [-4.44, 13.56]
**Subtotal (95% CI)**			**7.6%**	**8.45 [6.30, 10.60]**
Heterogeneity: Tau² = 3.75; Chi² = 12.13, df = 6 (P = 0.06); I² = 51%				
Test for overall effect: Z = 7.70 (P < 0.00001)				
**11.2.2 Multicenter**				
SIL001	9.1	0.67983266	1.9%	9.10 [7.77, 10.43]
SIL002	11.7	0.66333333	1.9%	11.70 [10.40, 13.00]
SIL003	9.3	0.71435285	1.9%	9.30 [7.90, 10.70]
SIL009	8.8	0.90647888	1.8%	8.80 [7.02, 10.58]
SIL011	8.9	1.00020314	1.8%	8.90 [6.94, 10.86]
SIL014	9.17	0.97966168	1.8%	9.17 [7.25, 11.09]
SIL016	8	0.89960563	1.8%	8.00 [6.24, 9.76]
SIL017	8.7	0.59961949	2.0%	8.70 [7.52, 9.88]
SIL018	6.1	0.60008187	2.0%	6.10 [4.92, 7.28]
SIL022	9.5	0.31698117	2.1%	9.50 [8.88, 10.12]
SIL023	3.6	0.39019949	2.0%	3.60 [2.84, 4.36]
SIL026	2.1	0.5999588	2.0%	2.10 [0.92, 3.28]
SIL028	6.39	1.18793939	1.7%	6.39 [4.06, 8.72]
SIL030	5.4	0.59958707	2.0%	5.40 [4.22, 6.58]
SIL034	10.37	0.92024897	1.8%	10.37 [8.57, 12.17]
SIL035a	6.1	0.51925763	2.0%	6.10 [5.08, 7.12]
SIL035b	6.8	0.81661203	1.9%	6.80 [5.20, 8.40]
SIL53	11.2	1.46262421	1.5%	11.20 [8.33, 14.07]
TAD001	7.8	0.99393222	1.8%	7.80 [5.85, 9.75]
TAD004	9.4	0.95863335	1.8%	9.40 [7.52, 11.28]
TAD005	9.35	0.80414166	1.9%	9.35 [7.77, 10.93]
TAD006	9.3	0.80016665	1.9%	9.30 [7.73, 10.87]
TAD007	8.5	0.70502286	1.9%	8.50 [7.12, 9.88]
TAD008	7	0.71851699	1.9%	7.00 [5.59, 8.41]
TAD009	4.5	0.90264274	1.8%	4.50 [2.73, 6.27]
TAD01	8	0.79948976	1.9%	8.00 [6.43, 9.57]
TAD012	8.03	0.44876926	2.0%	8.03 [7.15, 8.91]
TAD015	7.3	0.49739114	2.0%	7.30 [6.33, 8.27]
TAD018	6.5	0.48989996	2.0%	6.50 [5.54, 7.46]
TAD02	6	0.78868662	1.9%	6.00 [4.45, 7.55]
TAD04	8	1.13863886	1.7%	8.00 [5.77, 10.23]
TAD06	5.6	1.01251693	1.8%	5.60 [3.62, 7.58]
TAD07	6.9	0.75012602	1.9%	6.90 [5.43, 8.37]
TAD08	9.3	0.60016134	2.0%	9.30 [8.12, 10.48]
TAD09	11.1	0.55882838	2.0%	11.10 [10.00, 12.20]
TAD10	9.8	0.40808442	2.0%	9.80 [9.00, 10.60]
TAD11	8.7	0.67618696	1.9%	8.70 [7.37, 10.03]
TAD16	5.3	0.50642857	2.0%	5.30 [4.31, 6.29]
TAD18	7.8	1.2	1.6%	7.80 [5.45, 10.15]
VAR005	10	0.39467611	2.0%	10.00 [9.23, 10.77]
VAR006	7.79	0.63463489	1.9%	7.79 [6.55, 9.03]
VAR007	10.9	0.81943603	1.9%	10.90 [9.29, 12.51]
VAR008	12.7	0.31544319	2.1%	12.70 [12.08, 13.32]
VAR018	8.6	0.54702374	2.0%	8.60 [7.53, 9.67]
VAR019	8.5	0.62757064	1.9%	8.50 [7.27, 9.73]
VAR020	8.95	1.39004796	1.5%	8.95 [6.23, 11.67]
VAR09	9	0.65652973	1.9%	9.00 [7.71, 10.29]
VAR10	7.8	0.67515839	1.9%	7.80 [6.48, 9.12]
VAR11	8.2	0.50823958	2.0%	8.20 [7.20, 9.20]
**Subtotal (95% CI)**			**92.4%**	**8.11 [7.42, 8.80]**
Heterogeneity: Tau² = 5.44; Chi² = 733.88, df = 48 (P < 0.00001); I² = 93%				
Test for overall effect: Z = 23.12 (P < 0.00001)				
**Total (95% CI)**			**100.0%**	**8.14 [7.48, 8.79]**
Heterogeneity: Tau² = 5.39; Chi² = 746.02, df = 55 (P < 0.00001); I² = 93%				
Test for overall effect: Z = 24.23 (P < 0.00001)				
Test for subgroup differences: Chi² = 0.09, df = 1 (P = 0.76), I² = 0%				

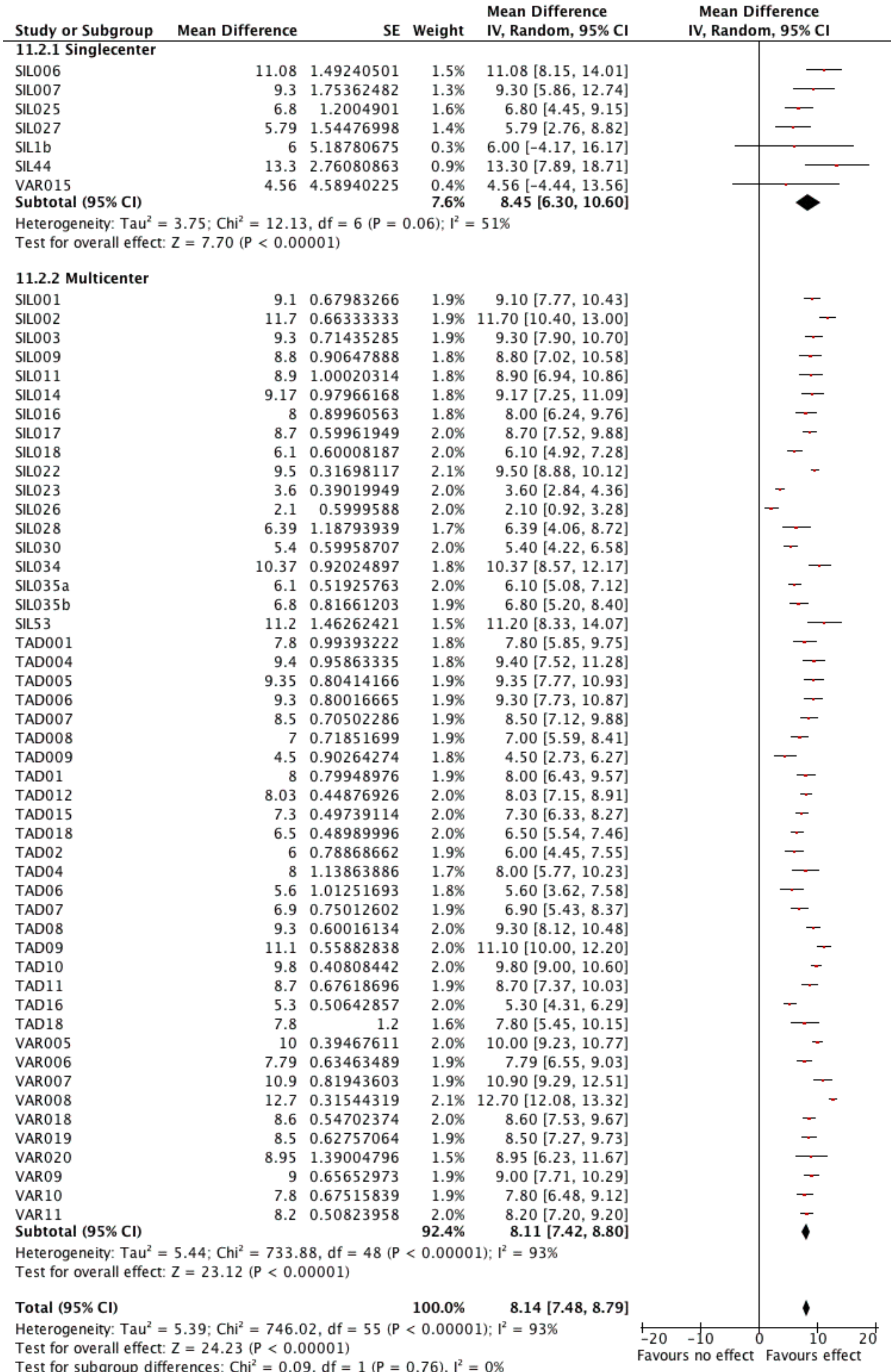

Forest plot of comparison: 11 IIEF-EF change scores: Single-center study, outcome: 11.2 Effect estimate intervention groups.

Figure 16 (Analysis 12.1)

Study or Subgroup	Mean Difference	SE	Weight	Mean Difference IV, Random, 95% CI
**12.1.1 Sildenafil**				
SIL001	2.3	0.6798897	2.0%	2.30 [0.97, 3.63]
SIL002	5.2	0.66312884	2.0%	5.20 [3.90, 6.50]
SIL003	3.6	0.71429868	1.9%	3.60 [2.20, 5.00]
SIL009	4.6	1.41138514	1.0%	4.60 [1.83, 7.37]
SIL011	3.4	1.0002526	1.5%	3.40 [1.44, 5.36]
SIL014	1.86	0.9617741	1.5%	1.86 [−0.03, 3.75]
SIL016	2.2	0.89950884	1.6%	2.20 [0.44, 3.96]
SIL017	1.4	0.59961949	2.1%	1.40 [0.22, 2.58]
SIL018	0.2	0.59965555	2.1%	0.20 [−0.98, 1.38]
SIL022	2.1	0.41837395	2.5%	2.10 [1.28, 2.92]
SIL023	2.2	0.38152839	2.5%	2.20 [1.45, 2.95]
SIL024	2.6	1.08010466	1.3%	2.60 [0.48, 4.72]
SIL025	1.4	1.0002526	1.5%	1.40 [−0.56, 3.36]
SIL026	−0.5	0.69990606	2.0%	−0.50 [−1.87, 0.87]
SIL027	0.92	2.07735223	0.5%	0.92 [−3.15, 4.99]
SIL028	1.24	1.24772593	1.1%	1.24 [−1.21, 3.69]
SIL030	1.1	0.69971892	2.0%	1.10 [−0.27, 2.47]
SIL034	2.66	1.06996831	1.4%	2.66 [0.56, 4.76]
SIL035a	0.2	0.59965555	2.1%	0.20 [−0.98, 1.38]
SIL035b	−0.9	0.69636364	2.0%	−0.90 [−2.26, 0.46]
SIL13	0.1	0.71429868	1.9%	0.10 [−1.30, 1.50]
SIL15	0	2.18	0.5%	0.00 [−4.27, 4.27]
SIL16	−1.2	0.94725968	1.5%	−1.20 [−3.06, 0.66]
SIL31	0.2	0.6729535	2.0%	0.20 [−1.12, 1.52]
SIL34	2.16	0.69636364	2.0%	2.16 [0.80, 3.52]
SIL35	4.56	0.73035234	1.9%	4.56 [3.13, 5.99]
SIL36	1.1	0.61215244	2.1%	1.10 [−0.10, 2.30]
SIL37	1.53	0.68587126	2.0%	1.53 [0.19, 2.87]
SIL44	1.7	1.27043518	1.1%	1.70 [−0.79, 4.19]
SIL53	2.5	1.54924372	0.9%	2.50 [−0.54, 5.54]
**Subtotal (95% CI)**			**50.5%**	**1.67 [1.10, 2.24]**
Heterogeneity: $Tau^2 = 1.75$; $Chi^2 = 121.92$, df = 29 (P < 0.00001); $I^2 = 76\%$				
Test for overall effect: Z = 5.76 (P < 0.00001)				
**12.1.2 Tadalafil**				
TAD001	0.1	1.14475367	1.3%	0.10 [−2.14, 2.34]
TAD005	0.87	0.77158927	1.8%	0.87 [−0.64, 2.38]
TAD006	2.3	1.60067954	0.8%	2.30 [−0.84, 5.44]
TAD01	−0.9	1.09985226	1.3%	−0.90 [−3.06, 1.26]
TAD012	0.49	0.78135299	1.8%	0.49 [−1.04, 2.02]
TAD015	3.3	0.73057328	1.9%	3.30 [1.87, 4.73]
TAD018	1.8	0.5100098	2.3%	1.80 [0.80, 2.80]
TAD02	1.1	1.0002526	1.5%	1.10 [−0.86, 3.06]
TAD03	0.1	0.78327589	1.8%	0.10 [−1.44, 1.64]
TAD04	2.6	0.95506746	1.5%	2.60 [0.73, 4.47]
TAD05	−1.3	1.28569386	1.1%	−1.30 [−3.82, 1.22]
TAD06	0.7	0.92697107	1.6%	0.70 [−1.12, 2.52]
TAD07	−0.2	1.08074954	1.3%	−0.20 [−2.32, 1.92]
TAD08	0.3	0.79963012	1.8%	0.30 [−1.27, 1.87]
TAD09	0.4	0.94285714	1.5%	0.40 [−1.45, 2.25]
TAD10	1.4	0.68914269	2.0%	1.40 [0.05, 2.75]
TAD11	2.4	0.69350381	2.0%	2.40 [1.04, 3.76]
TAD16	1.1	0.61529978	2.1%	1.10 [−0.11, 2.31]
TAD18	1	0.89924413	1.6%	1.00 [−0.76, 2.76]
**Subtotal (95% CI)**			**31.0%**	**1.05 [0.56, 1.55]**
Heterogeneity: $Tau^2 = 0.48$; $Chi^2 = 30.67$, df = 18 (P = 0.03); $I^2 = 41\%$				
Test for overall effect: Z = 4.14 (P < 0.0001)				
**12.1.3 Vardenafil**				
VAR005	2	0.67182644	2.0%	2.00 [0.68, 3.32]
VAR006	2.05	0.47433528	2.4%	2.05 [1.12, 2.98]
VAR007	1.6	0.99406573	1.5%	1.60 [−0.35, 3.55]
VAR008	1.69	0.7199074	1.9%	1.69 [0.28, 3.10]
VAR015	3.63	4.85004527	0.1%	3.63 [−5.88, 13.14]
VAR018	1.4	0.5230701	2.3%	1.40 [0.37, 2.43]
VAR019	1.4	0.48540962	2.4%	1.40 [0.45, 2.35]
VAR020	0.48	0.96670525	1.5%	0.48 [−1.41, 2.37]
VAR09	1.6	0.64250837	2.1%	1.60 [0.34, 2.86]
VAR11	0.5	0.40379108	2.5%	0.50 [−0.29, 1.29]
**Subtotal (95% CI)**			**18.6%**	**1.37 [0.99, 1.74]**
Heterogeneity: $Tau^2 = 0.00$; $Chi^2 = 9.03$, df = 9 (P = 0.43); $I^2 = 0\%$				
Test for overall effect: Z = 7.10 (P < 0.00001)				
**Total (95% CI)**			**100.0%**	**1.42 [1.08, 1.75]**
Heterogeneity: $Tau^2 = 1.00$; $Chi^2 = 166.72$, df = 58 (P < 0.00001); $I^2 = 65\%$				
Test for overall effect: Z = 8.31 (P < 0.00001)				
Test for subgroup differences: $Chi^2 = 2.57$, df = 2 (P = 0.28), $I^2 = 22.3\%$				

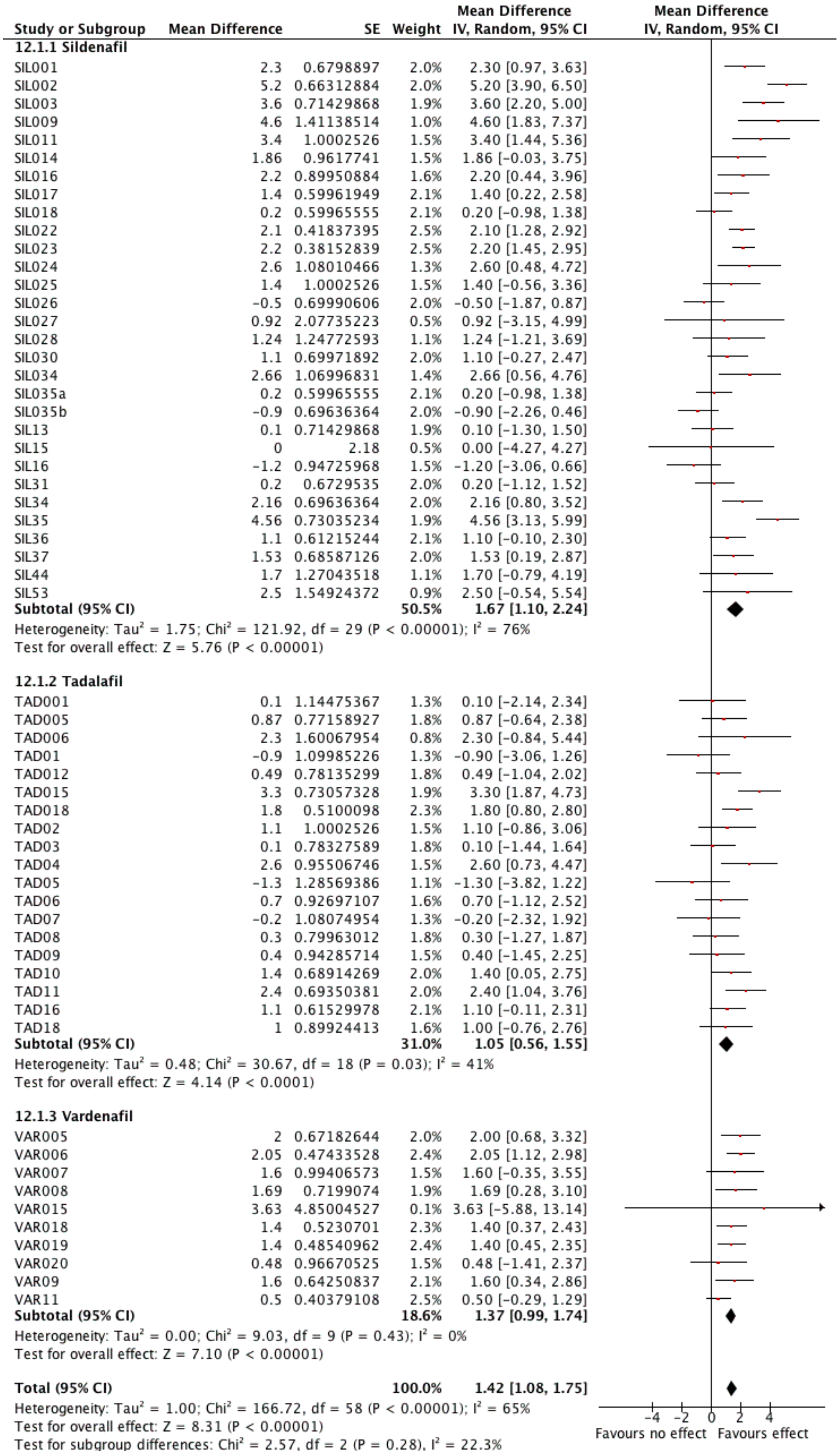

Forest plot of comparison: 12 IIEF-EF change scores: Type of PDE-5 inhibitor, outcome: 12.1 Effect estimate placebo groups.

Figure 17 (Analysis 12.2)

Study or Subgroup	Mean Difference	SE	Weight	Mean Difference IV, Random, 95% CI
**12.2.1 Sildenafil**				
SIL001	9.1	0.67983266	1.8%	9.10 [7.77, 10.43]
SIL002	11.7	0.66333333	1.8%	11.70 [10.40, 13.00]
SIL003	9.3	0.71435285	1.8%	9.30 [7.90, 10.70]
SIL009	8.8	0.90647888	1.7%	8.80 [7.02, 10.58]
SIL011	8.9	1.00020314	1.6%	8.90 [6.94, 10.86]
SIL014	9.17	0.97966168	1.6%	9.17 [7.25, 11.09]
SIL016	8	0.89960563	1.7%	8.00 [6.24, 9.76]
SIL017	8.7	0.59961949	1.8%	8.70 [7.52, 9.88]
SIL018	6.1	0.60008187	1.8%	6.10 [4.92, 7.28]
SIL022	9.5	0.31698117	1.9%	9.50 [8.88, 10.12]
SIL023	3.6	0.39019949	1.9%	3.60 [2.84, 4.36]
SIL024	7.2	0.75046652	1.8%	7.20 [5.73, 8.67]
SIL025	6.8	1.2004901	1.5%	6.80 [4.45, 9.15]
SIL026	2.1	0.5999588	1.8%	2.10 [0.92, 3.28]
SIL027	5.79	1.54476998	1.3%	5.79 [2.76, 8.82]
SIL028	6.39	1.18793939	1.5%	6.39 [4.06, 8.72]
SIL030	5.4	0.59958707	1.8%	5.40 [4.22, 6.58]
SIL034	10.37	0.92024897	1.7%	10.37 [8.57, 12.17]
SIL035a	6.1	0.60008187	1.8%	6.10 [4.92, 7.28]
SIL035b	6.8	0.67618696	1.8%	6.80 [5.47, 8.13]
SIL13	9.5	0.68445024	1.8%	9.50 [8.16, 10.84]
SIL15	7	1.15828321	1.6%	7.00 [4.73, 9.27]
SIL16	8.4	0.86081409	1.7%	8.40 [6.71, 10.09]
SIL31	11.1	0.68256437	1.8%	11.10 [9.76, 12.44]
SIL34	11.76	0.67618696	1.8%	11.76 [10.43, 13.09]
SIL35	10.66	0.72081772	1.8%	10.66 [9.25, 12.07]
SIL36	10	0.71619815	1.8%	10.00 [8.60, 11.40]
SIL37	10.85	0.66333333	1.8%	10.85 [9.55, 12.15]
SIL44	13.3	2.76080863	0.8%	13.30 [7.89, 18.71]
SIL53	11.2	1.46262421	1.4%	11.20 [8.33, 14.07]
**Subtotal (95% CI)**			**50.7%**	**8.37 [7.37, 9.37]**
Heterogeneity: $Tau^2 = 7.00$; $Chi^2 = 447.95$, df = 29 ($P < 0.00001$); $I^2 = 94\%$				
Test for overall effect: Z = 16.39 ($P < 0.00001$)				
**12.2.2 Tadalafil**				
TAD001	7.8	0.98163384	1.6%	7.80 [5.88, 9.72]
TAD005	9.35	0.80414166	1.7%	9.35 [7.77, 10.93]
TAD006	9.3	0.80016665	1.7%	9.30 [7.73, 10.87]
TAD01	8	0.79948976	1.7%	8.00 [6.43, 9.57]
TAD012	8.03	0.44876926	1.9%	8.03 [7.15, 8.91]
TAD015	7.3	0.49739114	1.9%	7.30 [6.33, 8.27]
TAD018	6.5	0.48989996	1.9%	6.50 [5.54, 7.46]
TAD02	6	1.00020314	1.6%	6.00 [4.04, 7.96]
TAD03	7.3	1.09601551	1.6%	7.30 [5.15, 9.45]
TAD04	8	1.13863886	1.6%	8.00 [5.77, 10.23]
TAD05	7.7	0.95310804	1.7%	7.70 [5.83, 9.57]
TAD06	5.6	1.01251693	1.6%	5.60 [3.62, 7.58]
TAD07	6.9	0.75012602	1.8%	6.90 [5.43, 8.37]
TAD08	9.3	0.60016134	1.8%	9.30 [8.12, 10.48]
TAD09	11.1	0.55882838	1.8%	11.10 [10.00, 12.20]
TAD10	9.8	0.40808442	1.9%	9.80 [9.00, 10.60]
TAD11	8.7	0.67618696	1.8%	8.70 [7.37, 10.03]
TAD16	5.3	0.50642857	1.9%	5.30 [4.31, 6.29]
TAD18	7.8	1.2	1.5%	7.80 [5.45, 10.15]
**Subtotal (95% CI)**			**33.0%**	**7.93 [7.15, 8.70]**
Heterogeneity: $Tau^2 = 2.34$; $Chi^2 = 113.38$, df = 18 ($P < 0.00001$); $I^2 = 84\%$				
Test for overall effect: Z = 20.11 ($P < 0.00001$)				
**12.2.3 Vardenafil**				
VAR005	10	0.66305586	1.8%	10.00 [8.70, 11.30]
VAR006	7.79	0.63463489	1.8%	7.79 [6.55, 9.03]
VAR007	10.9	0.81943603	1.7%	10.90 [9.29, 12.51]
VAR008	12.7	0.31544319	1.9%	12.70 [12.08, 13.32]
VAR015	4.56	4.58940225	0.4%	4.56 [−4.44, 13.56]
VAR018	8.6	0.54702374	1.8%	8.60 [7.53, 9.67]
VAR019	8.5	0.62757064	1.8%	8.50 [7.27, 9.73]
VAR020	8.95	1.39004796	1.4%	8.95 [6.23, 11.67]
VAR09	9	0.65652973	1.8%	9.00 [7.71, 10.29]
VAR11	8.2	0.50823958	1.9%	8.20 [7.20, 9.20]
**Subtotal (95% CI)**			**16.3%**	**9.32 [7.88, 10.77]**
Heterogeneity: $Tau^2 = 4.48$; $Chi^2 = 115.03$, df = 9 ($P < 0.00001$); $I^2 = 92\%$				
Test for overall effect: Z = 12.66 ($P < 0.00001$)				
**Total (95% CI)**			**100.0%**	**8.37 [7.73, 9.00]**
Heterogeneity: $Tau^2 = 5.43$; $Chi^2 = 777.88$, df = 58 ($P < 0.00001$); $I^2 = 93\%$				
Test for overall effect: Z = 25.79 ($P < 0.00001$)				
Test for subgroup differences: $Chi^2 = 2.84$, df = 2 (P = 0.24), $I^2 = 29.5\%$				

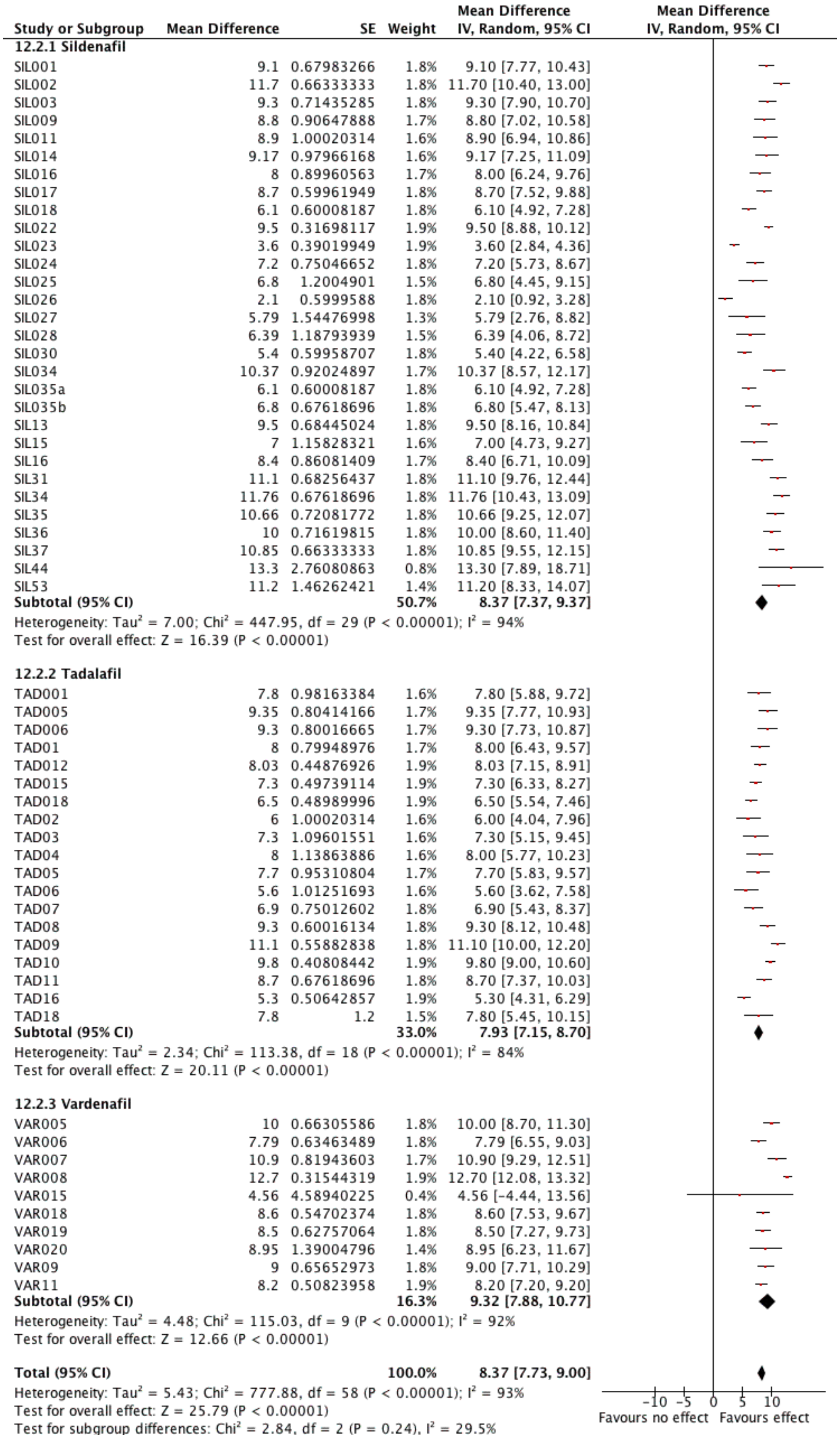

Forest plot of comparison: 12 IIEF-EF change scores: Type of PDE-5 inhibitor, outcome: 12.2 Effect estimate intervention groups.

Figure 18 (Analysis 13.1)

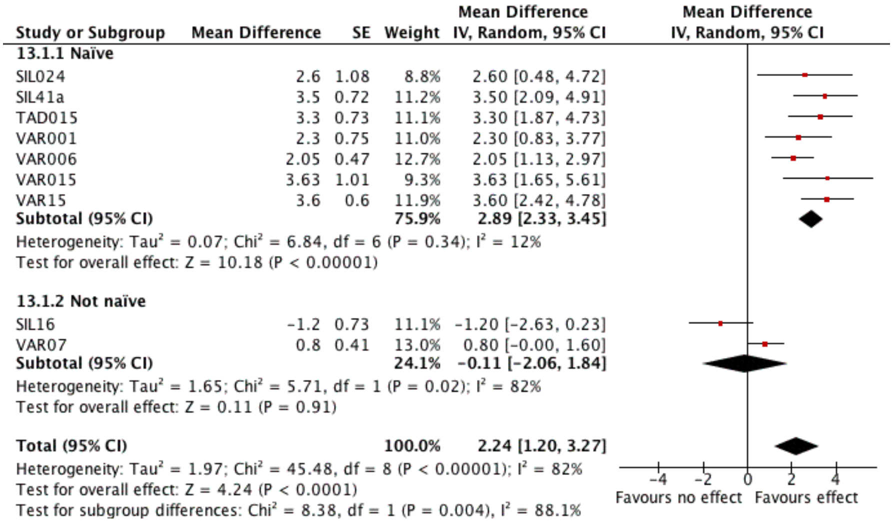

Forest plot of comparison: 13 IIEF-EF change scores: Prior experience with the intervention, outcome: 13.1 Effect estimate placebo groups.

Figure 19 (Analysis 13.2)

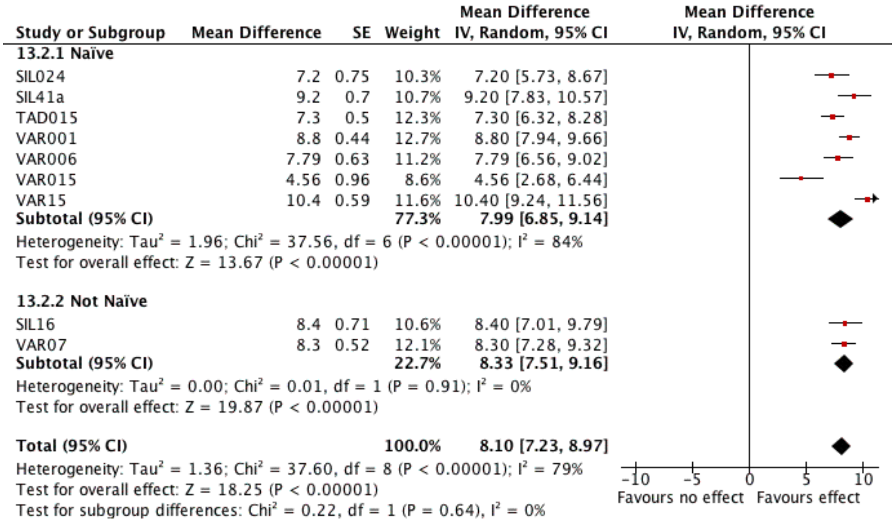

Forest plot of comparison: 13 IIEF-EF change scores: Prior experience with the intervention, outcome: 13.2 Effect estimate intervention groups.

Sources of support

Internal sources

- No sources of support provided

External sources

- No sources of support provided

Feedback

Appendices
